# Aberrant activation of non-coding RNA targets of transcriptional elongation complexes contributes to TDP-43 toxicity

**DOI:** 10.1038/s41467-018-06543-0

**Published:** 2018-10-23

**Authors:** Chia-Yu Chung, Amit Berson, Jason R. Kennerdell, Ashley Sartoris, Travis Unger, Sílvia Porta, Hyung-Jun Kim, Edwin R. Smith, Ali Shilatifard, Vivianna Van Deerlin, Virginia M.-Y. Lee, Alice Chen-Plotkin, Nancy M. Bonini

**Affiliations:** 1 Department of Biology, University of Pennsylvania, Philadelphia, PA 19104 USA; 2 Cell and Molecular Biology Graduate Group, Perelman School of Medicine, Philadelphia, PA 19104 USA; 3 Department of Neurology, Perelman School of Medicine, Philadelphia, PA 19104 USA; 4 Department of Pathology and Laboratory Medicine, Perelman School of Medicine, Philadelphia, PA 19104 USA; 5 Department of Biochemistry and Molecular Genetics, Feinberg School of Medicine, Northwestern University, Chicago, IL 60611 USA; 6 Present Address: Department of Neural Development and Disease, Korea Brain Research Institute (KBRI), Daegu, 41068 South Korea

## Abstract

TDP-43 is the major disease protein associated with amyotrophic lateral sclerosis (ALS) and frontotemporal lobar degeneration with ubiquitinated inclusions (FTLD-TDP). Here we identify the transcriptional elongation factor Ell—a shared component of little elongation complex (LEC) and super elongation complex (SEC)—as a strong modifier of TDP-43-mediated neurodegeneration. Our data indicate select targets of LEC and SEC become upregulated in the fly ALS/FTLD-TDP model. Among them, U12 snRNA and a stress-induced long non-coding RNA *Hsrω*, functionally contribute to TDP-43-mediated degeneration. We extend the findings of *Hsrω*, which we identify as a chromosomal target of TDP-43, to show that the human orthologue Sat III is elevated in a human cellular disease model and FTLD-TDP patient tissue. We further demonstrate an interaction between TDP-43 and human ELL2 by co-immunoprecipitation from human cells. These findings reveal important roles of Ell-complexes LEC and SEC in TDP-43-associated toxicity, providing potential therapeutic insight for TDP-43-associated neurodegeneration.

## Abstract (web summary)

TDP-43 is associated with amyotrophic lateral sclerosis (ALS) and frontotemporal lobar degeneration with ubiquitinated inclusions (FTD-TDP). Here, the authors identify the transcriptional elongation factor Ell as a strong modifier of TDP-43-mediated neurodegeneration through the Ell transcriptional elongation complexes LEC and SEC.

## Introduction

Amyotrophic lateral sclerosis (ALS; OMIM no. 105400) is the most common motor neuron disease, resulting from loss of motor neurons in the motor cortex, brainstem, and spinal cord, whereas frontotemporal dementia (FTD; OMIM no. 600274) is characterized by progressive changes in behavior, personality, and/or language due to gradual deterioration of the frontal and temporal lobes. Despite differences in primary sites of neurodegeneration, these two diseases share neuropathological and genetic commonalities, as well as clinical overlap^1,2^. TAR DNA-binding protein 43 (TDP-43) is the major component of inclusion bodies in most ALS and in half of FTD known as frontotemporal lobar degeneration with ubiquitinated inclusions (FTLD-TDP)^3,4^. Both depletion and upregulation of TDP-43 cause neuronal loss, indicating that TDP-43 levels are critical in the brain^5,6^. TDP-43 is an RNA-binding protein with many functions in RNA regulation and metabolism, and is one of a number of RNA-binding proteins associated with ALS and FTD^6–8^. This leads to an RNA dysregulation-centered view of TDP-43 disease mechanisms. TDP-43, however, also binds DNA and has been shown to regulate transcription^9–11^. The role of transcriptional dysfunction in TDP-43 proteinopathies remains largely unexplored.

*Drosophila melanogaster* has played an important role in elucidating roles of many genes in human neurological disease^12–14^. In *Drosophila*, expression of human TDP-43 leads to neuronal degeneration, shorten lifespan, and climbing defects, recapitulating fundamental disease features and serving as a platform to provide insight into underlying pathways and therapeutic targets^15,16^. To define novel disease mechanisms associated with TDP-43, we used *Drosophila* to screen for modifiers of toxicity. Our unbiased screen uncovered the transcription elongation factor *Ell* (also known as *Su(Tpl)*) gene as a novel and robust modulator. Ell is present in two complexes: little elongation complex (LEC) and super elongation complex (SEC)^17–19^. In *Drosophila*, LEC, containing Ell, Eaf, Ice1, and Ice2, regulates the initiation and elongation of Pol II-transcribed small nuclear RNA (snRNA) genes^19,20^. SEC is composed of Ell, Eaf, Ear, Lilli, and P-TEFb^19,21^. The kinase P-TEFb phosphorylates Pol II, leading to the release of paused Pol II into productive elongation. The composition and functions of both complexes are highly conserved in mammals^17,18,20^. Neither complex has been implicated in neurodegenerative disorders.

snRNAs together with a range of proteins form small nuclear ribonucleoproteins (snRNPs), which are essential components of the splicing machinery of the spliceosome. In both ALS and FTD patients, splicing changes have been observed by transcriptome and microarray analyses, implicating aberrant splicing as an important disease mechanism^22,23^. In ALS and human cell disease models with TDP-43 depletion, disruption of several snRNA levels has been reported, both higher^24^ and lower levels^25^, but the detailed mechanism and whether these alterations are functionally important in disease is unclear. In normal physiology, SEC activity has been shown to be specific to select conditions and genes: developmental genes under differentiation signals^26^, and heat shock genes upon stress^17,18^. The critical role of heat shock genes in neurodegenerative diseases has been revealed by studies on molecular chaperone and other proteins, which maintain proteostasis^27,28^. Among the heat shock genes, one distinct from others encodes no protein product but rather a long non-coding RNA (lncRNA), named Heat shock RNA omega (*Hsrω*). *Hsrω* is functionally required for normal development and the heat shock response in *Drosophila*, and is also involved in multiple other pathways potentially contributing to cellular homeostasis^29,30^. In humans, Satellite III repeat RNA (Sat III) is the functional orthologue of *Hsrω*^31^ and its role in neurodegenerative disease is unknown.

Here, we implicate Ell-associated transcriptional elongation complexes LEC and SEC as misregulated upon TDP-43 toxicity. We identify key non-coding RNA targets, U12 and *Hsrω*, as abnormally activated and functionally contributing to TDP-43-induced degeneration. Extension of these data to disease tissue and demonstration of the interaction between TDP-43 and one of the human orthologue of Ell, ELL2, implicates misregulation of human Ell orthologues as a contributor to TDP-43-associated pathologies.

## Results

### TDP-43 toxicity is mitigated by modulation of *Ell*

**Fig. 1:**
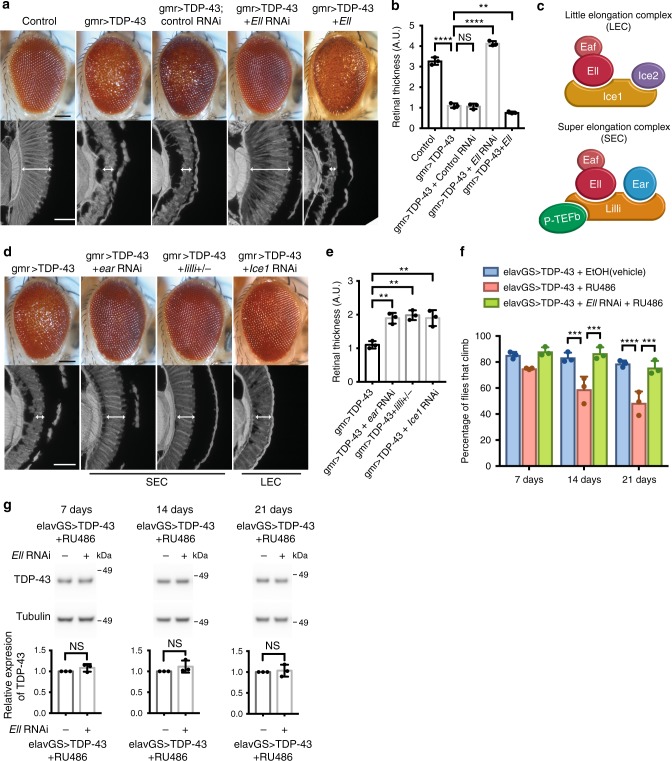
Components of LEC and SEC modulate TDP-43 toxicity. **a** TDP-43 expression causes eye degeneration that is suppressed by *Ell* RNAi and enhanced by *Ell* upregulation. Scale bars: external eye images (top), 100 μm; internal retinal section (bottom), 5 μm. Genotypes: control is *gmr-GAL4(YH3)/+*. gmr > TDP-43 is *UAS-TDP-43/+; gmr-GAL4(YH3)/+*. gmr > TDP-43 + Control RNAi is *UAS-TDP-43/+; gmr-GAL4(YH3)/UAS-Control.RNAi*^*JF01355*^. gmr > TDP-43 + *Ell* RNAi is *UAS-TDP-43/+; gmr-GAL4(YH3)/UAS-Ell.RNAi*^*HMS00277*^. gmr > TDP-43 + *Ell* is *UAS-TDP-43/+; gmr-GAL4(YH3)/UAS-Ell*^*P{EP}G4098*^. **b** Quantification of retina thickness related to **a**. Three flies of each genotypes were measured (*n* = 3). Bars represent mean (SD). **P < 0.01, ****P < 0.0001 (two-tailed unpaired Student’s *t*-test). Genotypes are the same as indicated in **a**. A.U., arbitrary units. **c** Schematic of LEC and SEC. **d** Downregulation of SEC components (*ear* and *lilli*), or LEC component (*Ice1*) suppresses TDP-43 toxicity. Scale bars: external eye (top), 100 μm; internal retina section (bottom), 5 μm. Genotypes: gmr > TDP-43 is *UAS-TDP-43/+*; *gmr-GAL4(YH3)/+*. gmr > TDP-43 + *ear* RNAi is *UAS-TDP-43/+; gmr-GAL4(YH3)/UAS-ear.RNAi*^*HMS00107*^. gmr > TDP-43 + *lilli+/-* is *UAS-TDP-43/lilli*^17–2^; *gmr-GAL4(YH3)/+*. gmr > TDP-43 + *Ice1* RNAi is *UAS-TDP-43/UAS-Ice1.RNAi* (SH09112.N from DRSC/TRiP); *gmr-GAL4(YH3)/+*. **e** Quantification of retina thickness related to **d**. Three flies of each genotypes were measured (*n* = 3). Bars represent mean (SD). **P < 0.01 (two-tailed unpaired Student’s *t*-test). Genotypes are the same as indicated in **d**. **f** TDP-43 expression in the adult neurons by *elavGS* causes climbing defects. Knockdown of *Ell* restores climbing ability. RU486 (8 mg/ml) was used to induce the expression of TDP-43 and *Ell* RNAi. EtOH was used as vehicle. Total of 100 flies were measured three times for each genotype at different time points. Bars represent mean (SD). ***P < 0.001, ****P < 0.0001 (two-way ANOVA followed by Tukey’s multiple comparison test). Significant differences are only indicated within the same time point. Genotypes: elavGS > TDP-43 is *elavGS-GAL4*, *UAS-TDP-43/*+. elavGS > TDP-43 + *Ell* RNAi is *elavGS-GAL4*, *UAS-TDP-43/UAS-Ell*.*RNAi* ^*HMS00277*^. **g** TDP-43 protein levels in heads from 7, 14 and 21-day flies are not altered by *Ell* RNAi. *n* = 3 biological replicates. Tubulin was used as internal control. Bars represent mean (SD). NS, not significant (two-tailed unpaired Student's *t*-test with Welch’s correction). Genotypes and RU486 treatment are as in **f**

Flies expressing human TDP-43 in the eye show retinal degeneration^15,16^. We employed these animals to screen for genes that enhanced or suppressed the degeneration of TDP-43. In the screen, 2933 fly lines with different genetic modifications were tested. Among them, 12 fly lines enhanced TDP-43 toxicity and 23 fly lines showed suppression, indicating a limited number of genes can modify TDP-43 toxicity. From this screen, we found that knockdown of the gene *Ell* strongly mitigated TDP-43-induced deterioration of the external eye and internal retina (Fig. 1a, b and Supplementary Fig. 1a). Furthermore, upregulation of *Ell* enhanced TDP-43 toxicity with more severe external eye and internal retinal degeneration (Fig. 1a, b and Supplementary Fig. 1a). These data indicated that *Ell* is a potent dose-dependent modifier of TDP-43, with *Ell* knockdown or upregulation on its own having no effect on eye morphology (Supplementary Fig. 1b). An RNA interference (RNAi) control line against luciferase did not mitigate TDP-43-mediated eye degeneration (Fig. 1a, b).

Ell protein is a shared component of two transcriptional elongation complexes: LEC and SEC (Fig. 1c). In order to investigate whether the suppression due to *Ell* knockdown was through one or the other of these complexes, we downregulated additional components of LEC and SEC in the presence of TDP-43. Reduction of SEC components *ear* and *lilli* partially suppressed the external and internal retinal deterioration conferred by TDP-43, as did reduction of LEC component *Ice1* (Fig. 1d, e and Supplementary Fig. 1a). The rescue effect of any of these components was not as strong upon depletion of the shared component *Ell*, suggesting that both LEC and SEC contribute to TDP-43 toxicity. Depletion of any of the components on their own had no effect on eye integrity (Supplementary Fig. 1b).

The levels of the TDP-43 protein were assessed by western immunoblot. These data indicated that enhancement by *Ell* upregulation or suppression by lowered levels of *Ell* or other components of LEC or SEC was not through modulating the levels of TDP-43 protein (Supplementary Fig. 1c). We further confirmed that the *GAL4-UAS* expression system was not impacted by any of these components by examining the levels of a control protein β-galactosidase (β-gal) (Supplementary Fig. 1d). Additional fly lines, including a genetic mutation of *Ell*, and two RNAi lines of *Ice1* with proper controls were tested and showed a consistent suppression effect on TDP-43-caused eye degeneration (Supplementary Fig. 1e).

To extend these studies to the nervous system generally, we expressed TDP-43 in all neurons in the adult animal using a conditional drug-inducible driver line. With expression in the adult fly brain induced by RU486, TDP-43 animals consistently show an age-associated decline in climbing ability, indicative of neural dysfunction (Fig. 1f, Supplementary Fig. 1f and ref. ^16^). Knockdown of *Ell*, although showing no effect on its own, restored climbing ability to normal without affecting TDP-43 protein levels (Fig. 1f, g and Supplementary Fig. 1g). We also assessed the suppression effect of *Ell* on TDP-43 toxicity in the nervous system by lifespan assays using the drug-inducible neuronal driver. Knockdown of *Ell* on its own caused a mild but statistically significant extension of lifespan, and downregulation of *Ell* caused a mild suppression of the TDP-43-shorten lifespan and a shift of the early stage of the lifespan curve (Supplementary Fig. 1h). Lifespan assays assessing the effect of *Ell* knockdown on TDP-43 toxicity were also tested by using a ubiquitous drug-inducible driver, showing a consistent result that *Ell* downregulation has some effect to mitigate TDP-43 toxicity (Supplementary Fig. 1I). These data indicate that increased activity of both Ell complexes LEC and SEC may contribute to TDP-43-mediated degeneration in the nervous system.

### LEC snRNA target U12 contributes to TDP-43 toxicity

**Fig. 2:**
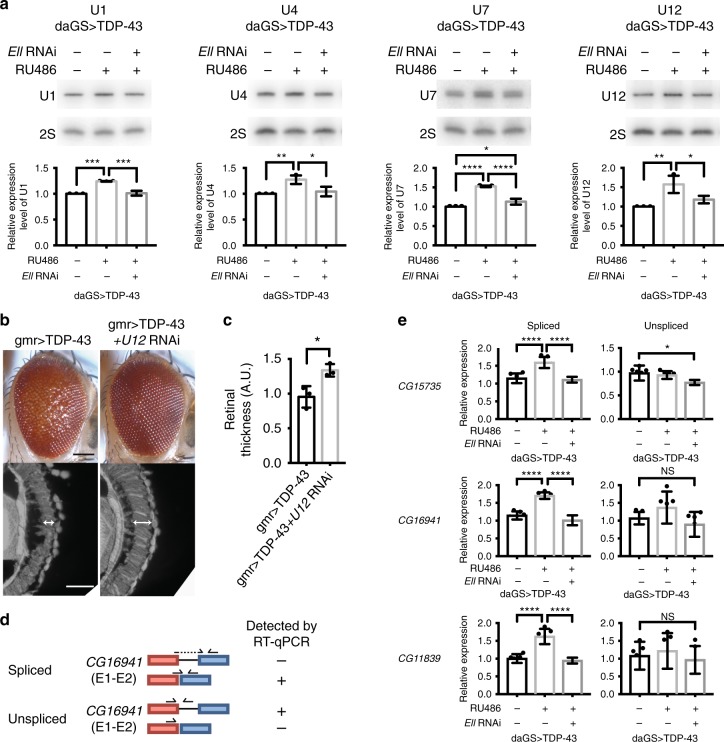
U12 snRNA is upregulated and a functional target of TDP-43. **a** Northern blot analysis shows that the level of snRNAs U1, U4, U7, and U12 are upregulated by TDP-43 expression driven by a ubiquitous drug-inducible promoter, *daGS*, in heads. *Ell* RNAi corrects the increased levels of snRNAs. RU486 (4 mg/ml) was used to induce the expression of TDP-43 and *Ell* RNAi for 8 days. 2S rRNA was the loading control. *n* = 3 biological replicates. Bars represent mean (SD). *P < 0.05, **P < 0.01, ***P < 0.001, ****P < 0.0001 (one-way ANOVA followed by Tukey’s multiple comparison test). Genotypes: daGS > TDP-43 is *daGS-GAL4/+; UAS-TDP-43/+*. daGS > TDP-43 + Ell RNAi is *daGS-GAL4/+; UAS-TDP-43/UAS-Ell.RNAi*^*HMS00277*^. **b** Knockdown of *U12* suppresses eye degeneration caused by TDP-43 toxicity. Scale bars: external eye (top), 100 μm; internal retina (bottom), 5 μm. Genotypes: gmr > TDP-43 is *UAS-TDP-43/+; gmr-GAL4(YH3)/+*. gmr > TDP-43 + *U12* RNAi is *UAS-TDP-43/UAS-snRNA:U12:73B.RNAi*^*HMC03841*^*; gmr-GAL4(YH3)/+*. **c** Quantification of retina thickness related to Fig. 2b. Three flies of each genotypes were measured (*n* = 3). Bars represent mean (SD). *P < 0.05 (two-tailed unpaired Student’s *t-*test). Genotypes are the same as indicated in **b** A.U., arbitrary units. **d** Schematic illustrates detection of spliced or unspliced products by RT-qPCR for a U12-type intron. **e** RT-qPCR analysis shows that spliced products of *CG15735*, *CG16941*, *CG11839* are upregulated by TDP-43 expression driven by *daGS* in fly heads, and downregulation of *Ell* rescues the increased levels, whereas the unspliced products are not changed significantly upon TDP-43 expression. RU486 (4 mg/ml) was used to induce expression for 8 days. mRNA levels were normalized to RpL32 mRNA. *n* = 6 biological replicates. Bars represent mean (SD). *P < 0.05, ****P < 0.0001 (one-way ANOVA followed by Tukey’s multiple comparison test). Genotypes are as indicated in **a**

To determine whether the activity and function of LEC and SEC are promoted by TDP-43, we assessed the downstream targets in the fly disease model. Targets of LEC are Pol II-transcribed snRNAs^19,20^. We therefore used northern blot analysis to assess the levels of snRNAs U1, U2, U4, U4atac, U5, U7, U11, and U12 in fly heads, with or without added TDP-43 driven by a ubiquitous drug-inducible driver. TDP-43 expression significantly increased levels of U1, U4, U7, and U12 (Fig. 2a and Supplementary Fig. 2b). Importantly, knockdown of *Ell* restored the levels of the elevated snRNAs back to normal without affecting TDP-43 expression (Fig. 2a and Supplementary Fig. 2a, b). These data suggest that misregulation of snRNA components may be a consequence of disrupted *Ell* function in the animals and contribute to TDP-43 toxicity.

To test the functional role of the snRNAs in TDP-43-induced degeneration, we determined whether downregulation of the elevated snRNAs could mitigate toxicity. We examined fly lines predicted to downregulate various snRNAs (U1, U4, U7, U12). There was little effect of fly lines directed to U1 and U4 (there are multiple copies of these snRNAs in the genome making interference challenging), and U7 depletion suppressed the external eye but did not suppress the internal deterioration. Although we cannot exclude the potential importance of U1, U4, and U7, we focused on U12. *U12* gene knockdown partially but consistently mitigated TDP-43-associated retinal disruption (Fig. 2b, c and Supplementary Fig. 2c), indicating that misregulation of U12 levels is functionally important to TDP-43 toxicity. We confirmed that U12 downregulation had no effect on TDP-43 protein, and that reduction of U12 on its own did not affect the eye (Supplementary Fig. 2d, e). U12 is a component of the minor spliceosome (U12-type spliceosome), indicating that targets of the minor spliceosome may be misregulated by TDP-43.

There are 18 genes containing a U12-type intron in *Drosophila*^32^. Total RNA from fly heads was prepared for reverse transcription quantitative PCR (RT-qPCR) with qPCR primer sets spanning the U12-regulated introns to assess the levels of spliced gene products (Fig. 2d). Among the 18 genes, six (*CG16941, CG11839, CG33108, CG11328, CG15735*, and *CG3294*) were upregulated with expression of TDP-43, with the other 12 genes not affected (Fig. 2e; Supplementary Fig. 2f, g), indicating increased U12 levels leads to elevation of specific minor spliceosome targets. Knockdown of *Ell* corrected the levels back to normal (Fig. 2e; Supplementary Fig. 2g), consistent with the ability of *Ell* downregulation to normalize the levels of U12 and protect from TDP-43 toxicity. To further determine whether the elevation resulted from transcription or splicing, we assessed the levels of the unspliced transcripts. The unspliced products of three genes, *CG33108*, *CG11328*, and *CG3294*, were also increased upon TDP-43 expression, indicating a change in total transcript levels (Supplementary Fig. 2g). The unspliced RNA levels of the three genes, *CG15735*, *CG16941* and *CG11839*, were not altered significantly by the presence of TDP-43, indicating that the splicing of these U12-regulated introns was increased (Fig. 2e). These data indicate that the U12-type spliceosome is abnormally activated in the fly disease model to cause disrupted regulation of selected downstream targets.

### TDP-43 and Lilli colocalize at the *Hsrω* lncRNA locus

**Fig. 3:**
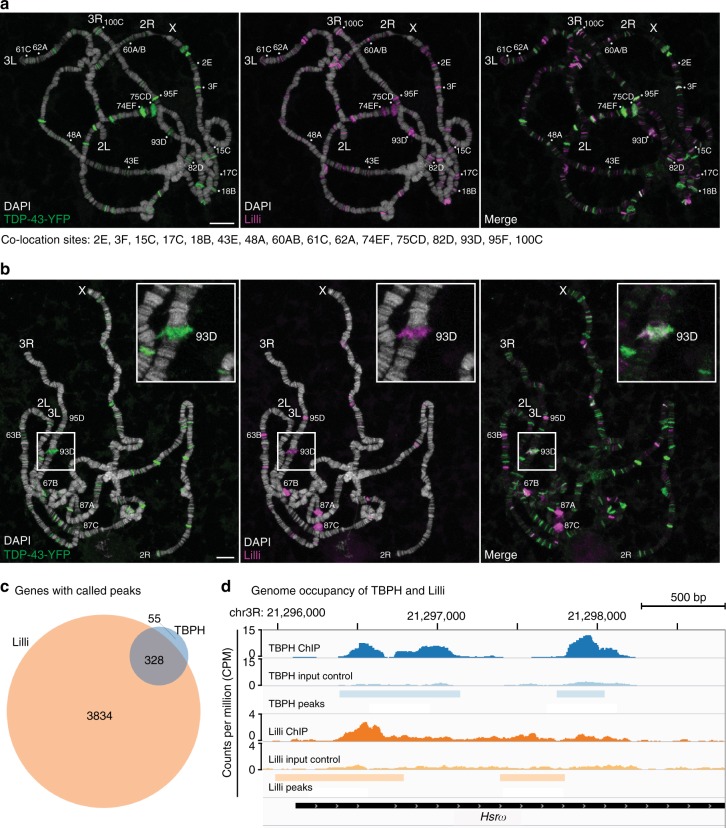
TDP-43 and Lilli colocalize at the 93D locus on polytene chromosomes. **a** Immunostainings of DAPI (white), TDP-43-YFP (green), and Lilli (magenta) show partial overlap of TDP-43-YFP and Lilli on chromosomes from salivary glands expressing TDP-43-YFP. Sixteen sites of consistent colocalization are indicated (bottom, see also Supplementary Table 1). **b** Upon heat shock, Lilli localizes to major heat shock loci and colocalizes with TDP-43-YFP at the 93D locus (enlarged at the upper right corner). See also Supplementary Table 2. For **a**, **b**, scale bars: 10 μm. Genotype: *sgs3-GAL4/UAS-TDP-43-YFP*. **c** Venn diagram illustrates the numbers of genes bound by TBPH and Lilli. **d** A ChIP-seq profile of TBPH (top, blue) and Lilli (bottom, orange) shows overlap at the *Hsrω* transcription start site. Bar intervals indicate called peaks in TBPH (light blue, top) and Lilli (light orange, bottom). The TBPH profile is one of four biological replicates that show called peaks at the *Hsrω* transcription start site

Ell is also a component of SEC and SEC activates transcriptional elongation, which is critical for genes involved in developmental signaling pathways and the stress response^17,18,26^. The SEC scaffold protein Lilli binds to and regulates specific targets on the chromosomes in *Drosophila* and human cells^17,18,26^. Recently, genome-wide chromatin immunoprecipitation (ChIP-seq) shows that *Drosophila* TDP-43, TBPH, associates with chromatin^11^. We therefore considered that TDP-43 may colocalize to the same genes as Lilli. To explore this, we expressed TDP-43 tagged with YFP in *Drosophila* salivary gland and assessed Lilli and TDP-43 localization on the polytene chromosomes by immunostaining. Under ambient temperature, we observed that TDP-43 binds to the polytene chromosomes with specificity (Fig. 3a). TDP-43 and Lilli each localized to ~115 sites (Fig. 3a). Most of these were euchromatin regions where active transcription usually occurs. Detailed analysis of the colocalization pattern indicated that TDP-43 and Lilli consistently overlapped at 16 sites across chromosomes X, 2R, 3R, and 3L (Fig. 3a and Supplementary Table 1). Although it is difficult to precisely identify the genes with colocalization of TDP-43 and Lilli due to the resolution of polytene bands, these data indicate partial overlap of TDP-43 and SEC targets in normal conditions; these genes may be important in toxicity.

Upon heat stress, SEC is recruited to major heat shock loci on the polytene chromosomes and, further, has been shown to regulate the expression of a major heat shock protein Hsp70^17^. Dysfunction of the heat shock response, which helps maintain proteostasis, is associated with TDP-43 toxicity^33–35^. We therefore probed whether TDP-43 colocalized to these targets of SEC. As noted^17^, with heat shock Lilli localizes to the major molecular chaperones at polytene chromosome sites 63B, 67B, 95D, 87A, and 87C and a unique heat shock locus, 93D that encodes the lncRNA *Hsrω* (Fig. 3b). When we examined colocalization with TDP-43, we noted some colocalization at 63B (encoding *Hsp83*) and 67B (encoding small heat shock genes) (Supplementary Table 2). Moreover, we observed robust and consistent colocalization at 93D in all polytene chromosome spreads examined (Fig. 3b and Supplementary Table 2); this was a site at which TDP-43 and Lilli also colocalized without added stress (Supplementary Table 1), suggesting that this stress-induced lncRNA may be a common target of TDP-43 and Lilli. We also assessed the colocalization of TDP-43 and Ell. Consistent with the results of immunostaining with Lilli, Ell also colocalized with TDP-43 at the 93D locus encoding *Hsrω* (Supplementary Fig. 3a). Precision nuclear run-on sequencing (PRO-seq) has shown that pol II is paused on *Hsrω*^36^, and chromatin staining shows that SEC components, Ell and Lilli, colocalize with elongating Pol II at the 93D locus after heat stress^17^, both indicating that *Hsrω* is a target of SEC.

Given the limited resolution of polytene chromosome staining, we analyzed the published ChIP-seq data for TBPH^11^ and Lilli^19^ to better define genes bound by both factors. This analysis showed that TBPH binds to 383 genes and Lilli binds to 4162 genes (Fig. 3c and Supplementary Data 1). Among them, 328 genes were bound by both factors, which comprises ~86% of the genes bound by TBPH and ~8% of the genes bound by Lilli (Fig. 3c). Consistent with the polytene data, *Hsrω* is a target of both TBPH and Lilli (Fig. 3d). We therefore considered that *Hsrω* might be a target of SEC that becomes misregulated by TDP-43.

### *Hsrω* functionally modulates TDP-43 toxicity

**Fig. 4:**
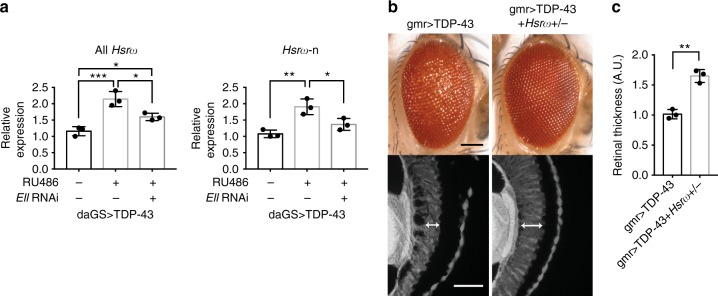
*Hsrω* is elevated and contributes to TDP-43 toxicity. **a** RT-qPCR analysis of fly heads shows that expression of TDP-43 driven by a drug-inducible promoter *daGS* leads to an increase of total *Hsrω* and *Hsrω-n*. Knockdown of *Ell* reduces the elevated levels of total *Hsrω* and *Hsrω-n*. RU486 (4 mg/ml) was used to induce expression for 4 days. Relative RNA levels were normalized to *Pgk*, *RpL32*, and *RpS20* mRNAs (geometric mean). *n* = 3 biological replicates. Bars represent mean (SD). *P < 0.05, **P < 0.01, ***P < 0.001 (one-way ANOVA followed by Tukey’s multiple comparison test). Genotype: daGS > TDP-43 is *daGS-GAL4/+*; *UAS-TDP-43/+*. daGS > TDP-43 + *Ell* RNAi is *daGS-GAL4/+; UAS-TDP-43/UAS-Ell.RNAi*^*HMS00277*^. **b** Images of external eyes (top) and internal retina (bottom) show that loss of one copy of *Hsrω* ameliorates TDP-43-caused eye degeneration. Scale bars: external eye, 100 μm; internal retina section, 5 μm. Genotypes: gmr > TDP-43 is *UAS-TDP-43/+; gmr-GAL4(YH3)/+*. gmr > TDP-43 + *Hsrω+/-* is *UAS-TDP-43/+; gmr-GAL4(YH3)/Hsrω*^66^. **c** Quantification of retina thickness related to Fig. 4b. Three flies of each genotypes were measured (*n* = 3). Bars represent mean (SD). **P < 0.01 (two-tailed unpaired Student’s *t*-test). Genotypes are the same as indicated in **b**. A.U., arbitrary units

The 93D locus encodes nuclear and cytoplasmic lncRNAs referred to as *Hsrω-n* and *Hsrω-c*, respectively, both of which are induced by stress. Upon heat stress, *Hsrω-n* transcripts are upregulated at the 93D site^29^, with a balanced level of *Hsrω* being critical for the survival and recovery of flies following heat stress^30^. Given the striking colocalization of TDP-43 and Lilli at the *Hsrω* locus, we considered that dysregulation of *Hsrω* may occur with TDP-43. RNA from fly heads in the presence or absence of added TDP-43 expression was extracted for RT-qPCR and the levels of total *Hsrω* (*Hsrω-c* and *Hsrω-n*), and *Hsrω-n* were determined. Total *Hsrω* and *Hsrω-n* were increased ~2-fold upon TDP-43 expression (Fig. 4a). Importantly, this misregulation was reduced toward normal levels by *Ell* knockdown (Fig. 4a). These data indicate that *Hsrω* may be a functional target of SEC activity that contributes to TDP-43-associated degeneration.

To assess whether misregulation of *Hsrω* is functionally important to TDP-43 toxicity, we reduced the levels of *Hsrω* in animals expressing TDP-43. Reduction of *Hsrω* by placing animals expressing TDP-43 in *trans* to an *Hsrω* null mutation partially mitigated TDP-43-associated external and internal retinal deterioration (Fig. 4b, c). This was not associated with changed levels of TDP-43 protein, and loss of one copy of *Hsrω* on its own had no effect (Supplementary Fig. 4a, b). We also confirm the suppression effect of *Hsrω* downregulation on TDP-43-mediated eye degeneration by a RNAi line with proper controls (Supplementary Fig. 4c). The effect of lacking one copy of *Hsrω* on the climbing defect caused by TDP-43 was unable to be determined because reduction of *Hsrω* on its own caused a decrease in climbing ability (data not shown). Taken together, these findings on the colocalization of TDP-43 and Lilli at the *Hsrω* gene locus, on elevated levels of *Hsrω* RNAs in TDP-43-expressing animals, and on mitigation of TDP-43-induced degeneration by modulation of *Hsrω* levels suggest that the stress-induced lncRNA *Hsrω* is a functional target of TDP-43 that contributes to TDP-43-mediated degeneration.

### Sat III is upregulated in a cellular model and in patient tissue

These data on *Hsrω* raised the possibility that the functional orthologue of *Hsrω* in humans, the stress-induced Satellite III repeat RNA (Sat III)^31^, may become misregulated in disease and contribute to toxicity. Although not sharing precise sequence similarity, *Hsrω* and Sat III transcripts share functional features and regulatory mechanisms: they are repeats and non-coding in nature; they are induced and accumulate at the site of synthesis upon stress, and associate with heterogeneous nuclear ribonucleoproteins (hnRNPs) and other RNA-processing factors; they are both Pol II-dependent transcripts^29,31,37^. Moreover, although Sat III is essential for cell survival after heat shock, upregulation of Sat III promotes cell death and acute senescence in various cell models^38,39^. We therefore investigated whether Sat III transcripts became misregulated by TDP-43.

**Fig. 5:**
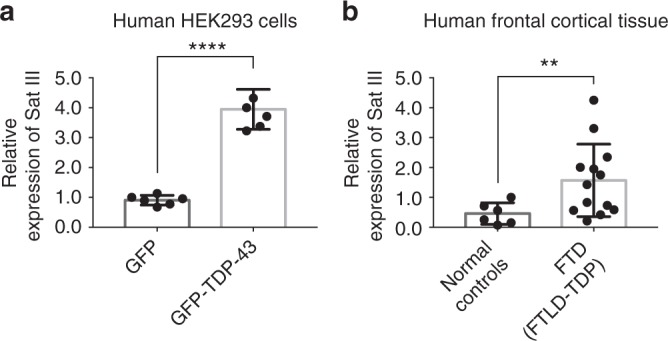
Sat III is upregulated in a human cell model and FTD patient tissue. **a** RT-qPCR analysis shows that the levels of Sat III are increased in HEK293 cells expressing GFP-TDP-43 after 6-day induction. Relative RNA levels were normalized to *GAPDH* and *ACTB* mRNAs (geometric mean). *n* = 6 biological replicates. Bars represent mean (SD). ****P *<* 0.0001 (two-tailed unpaired Student’s *t*-test with Welch’s correction). **b** RT-qPCR analysis shows that the levels of Sat III are increased significantly in frontal cortex of FTD patients compared with normal frontal cortex controls. Relative RNA levels were normalized to *GAPDH* and *ACTB* mRNAs (geometric mean). All FTD patients had TDP-43 pathology (FTLD-TDP). Case numbers and details are as indicated in Table 1. Bars represent mean (SD). **P *<* 0.01 (two-tailed unpaired Student’s *t*-test with Welch’s correction)

To assess Sat III levels in mammalian cells, we used human embryonic kidney 293 (HEK293) cells expressing doxycycline-induced green fluorescent protein (GFP)-tagged TDP-43 (GFP-TDP-43) or GFP alone. RNA was prepared following 6 days of induction, and the levels of Sat III transcripts were examined by RT-qPCR. We compared Sat III levels in cells expressing GFP-TDP-43 to control cells expressing GFP. TDP-43 expression led to a ~4-fold increase in the steady-state level of Sat III (Fig. 5a). The Sat III primers were validated by heat shock induction of Sat III followed by RT-qPCR analysis (Supplementary Fig. 5). Thus, the human counterpart of *Hsrω* became misregulated upon aberrant TDP-43 expression in cells.

These findings raised the possibility that Sat III may become dysregulated in human disease. To address this, we used RNA prepared from frontal cortex of 13 FTD patients (six sporadic or with unknown family history and seven patients with a family history of FTD and/or a disease-associated mutation) and six clinically normal controls (Fig. 5b). All of the FTD patients had TDP-43 pathology (FTLD-TDP)^40,41^. In accordance with our studies in the fly and human cells, the results showed that the levels of Sat III transcripts were significantly higher in the frontal cortex of FTD patients compared with controls (Fig. 5b). Case numbers and details are indicated in Table 1. Taken together with the functional data from the fly, misregulation of the stress-induced lncRNA Sat III may be a contributor to TDP-43-associated disease.

**Table 1:** Human brain samples

		Number
Normal controls	No mutation identified	6
FTD (FTLD-TDP)	No mutation identified	6
	GRN mutation	5
	C9Orf72 mutation	1
	VCP mutation	1

### TDP-43 and ELL2 proteins interact

**Fig. 6:**
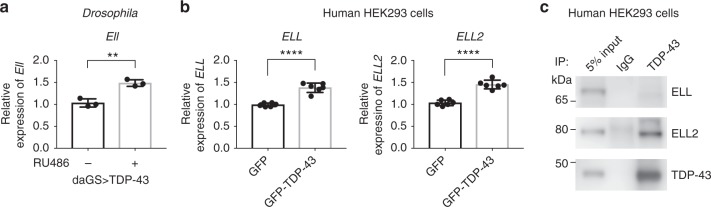
TDP-43 interacts with ELL2 in human cells. **a** RT-qPCR analysis of fly heads shows that expression of TDP-43 driven by *daGS* leads to an increase of *Ell* mRNA. RU486 (4 mg/ml) was used to induce expression for 4 days. Relative RNA levels were normalized to *Pgk*, *RpL32*, and *RpS20* mRNAs (geometric mean). *n* = 3 biological replicates. Bars represent mean (SD). **P < 0.01 (two-tailed unpaired Student’s *t*-test). Genotypes are the same as indicated in Fig. 4a. **b** RT-qPCR analysis shows that the mRNA levels of *ELL* and *ELL2* are increased in HEK293 cells expressing GFP-TDP-43 compared with cells expressing GFP after 6-day induction. Relative RNA levels were normalized to *GAPDH* and *ACTB* mRNAs (geometric mean). *n* = 6 biological replicates. Bars represent mean (SD). ****P *<* 0.0001 (two-tailed unpaired Student’s *t*-test). **c** IP with anti-TDP-43 antibody or mouse IgG as negative control followed by immunoblotting studies with anti-ELL, ELL2, or TDP-43 antibody show that TDP-43 interacts with ELL2. The co-IP assays were repeated independently four times and show consistent results (a different ELL2 antibody was used for detection for two repeats of the experiments)

To further dissect the relationship between Ell and TDP-43 and define additional mechanisms by which Ell may contribute to TDP-43-mediated neurodegeneration, we assessed whether Ell levels were upregulated in disease models; upregulation of Ell could lead to increase levels of downstream targets and in this manner contribute to TDP-43-mediated neurodegeneration. RNA levels of fly *Ell* were examined by RT-qPCR using RNA extracted from fly heads in the presence or absence of added TDP-43. These data showed that *Ell* is elevated ~50% upon TDP-43 expression (Fig. 6a). We next examined human ELL family proteins in the HEK293 cell model. In mammals, there are three proteins in the ELL family: ELL, ELL2 and ELL3^21^, with ELL3 being the most distinct based on sequence (~50% identity) and enriched in testis^42^. All three ELL proteins can be pulled down with factors in LEC and SEC^17,19^. We examined the RNA levels of *ELL* and *ELL2* in cells expressing GFP-TDP-43 or GFP control by RT-qPCR. The results are consistent with the fly data, showing that TDP-43 expression led to a 50% increase of *ELL* and *ELL2* levels (Fig. 6b). However, when we assessed protein levels by western immunoblot, the results indicated that the levels of ELL and ELL2 were not changed (Supplementary Fig. 6a). We also examined *ELL* and *ELL2* by RT-qPCR using RNA prepared from frontal cortex of the 13 FTD patients with TDP-43 pathology; expression levels of *ELL* and *ELL2* were not changed significantly compared with controls (Supplementary Fig. 6a). These results, together with the data showing that upregulation of *Ell* on its own does not cause eye degeneration (Fig. 1a and Supplementary Fig. 1b), indicate that mechanisms beyond simply a global increase in levels of *Ell* may be involved in TDP-43-associated toxicity.

The polytene chromosome immunostaining showed that TDP-43 colocalized with Ell and Lilli at the 93D locus (Fig. 3a and Supplementary Fig. 3a), indicating that interactions between the proteins might occur. We thus assessed whether endogenous TDP-43 interacts with ELL or ELL2 in nuclear extracts of HEK293 cells using co-immunoprecipitation (co-IP). TDP-43 was immunoprecipitated by an anti-TDP-43 antibody (Fig. 6c), and western blots then probed for presence of co-IPed ELL or ELL2. ELL was not detected; however, ELL2 was co-IPed together with TDP-43 (Fig. 6c). This was specific because ELL2 was not co-IPed with IgG control. These data indicate that TDP-43 interacts with ELL2, suggesting that this interaction may contribute to dysfunction of ELL-associated complexes upon aberrant TDP-43 function.

## Discussion

**Fig. 7:**
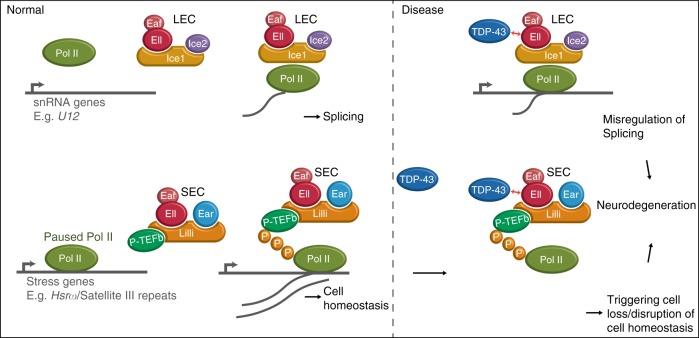
TDP-43 promotes the levels of the targets of LEC and SEC contributing to neurodegeneration. A model for TDP-43 toxicity associated with LEC and SEC. LEC regulates the transcription of snRNAs, forming spliceosomes, and SEC regulates stress response genes, maintaining cell homeostasis. In the disease state, TDP-43 promotes the levels of LEC and SEC targets through interactions with Ell in fly/ELL2 in human cells, including U12 and Hsrω/Sat III, leading to misregulation of splicing, cell loss, and disruption of cell homeostasis and contributes to neurodegeneration

Misregulation of several transcriptional elongation factors has been reported in diseases, including viral pathogenesis and cancer^43,44^, yet these factors have not been implicated in neurodegenerative disorders. Here we report that Ell and Ell-containing transcriptional elongation complexes LEC and SEC are novel modifiers of TDP-43 toxicity. The levels of several LEC target snRNAs including U12 are upregulated by TDP-43 proteinopathy. Our data suggest that the U12-type spliceosome is abnormally activated and contributes to toxicity. Through chromatin immunostaining, we identify a key target of SEC, a stress-induced lncRNA *Hsrω*, as aberrantly elevated and functionally important to TDP-43-mediated degeneration. In addition, we show that the levels of the human orthologue of *Hsrω*, Sat III, is increased in a human cell model and in FTLD-TDP frontal cortex tissue, indicating that Sat III dysfunction may contribute to TDP-43-associated disease. Finally, we demonstrate TDP-43 interacts with the one of the human orthologues of Ell, ELL2, indicating that the aberrant elevation of LEC and SEC activity in disease may be promoted through the interactions between TDP-43 and ELL2 (Fig. 7). These findings highlight the critical roles of LEC and SEC in TDP-43-mediated pathologies, and highlight that approaches to normalize the activity of human orthologues of the shared component Ell may be of therapeutic benefit.

Our data suggest a model whereby SEC and LEC contribute to TDP-43-mediated degeneration in parallel (Fig. 7). We find that that depletion of the shared component *Ell* led to nearly full suppression of TDP-43-mediated eye degeneration, whereas, by contrast, downregulation of LEC- and SEC-specific components or downstream targets U12 and *Hsrω* showed only partial suppression, despite the downregulation effect for the genes being robust. In our study, we identified important targets that are functionally involved in TDP-43-mediated degeneration, but we do not exclude the possibility that there may be other targets regulated by LEC and SEC that are important in the disease. Furthermore, we show that upregulation of the shared component *Ell* enhances TDP-43 degeneration, and the abnormally elevated levels of LEC and SEC targets caused by TDP-43 toxicity can be rescued by *Ell* depletion. Our study demonstrates that TDP-43-mediated degeneration can be alleviated by decreasing the activities of LEC and SEC, which is predicted to lead to reduction of elongating Pol II on downstream key targets and thus lower their levels.

As an RNA-binding protein, TDP-43 has been demonstrated to regulate targets through direct binding^6,7,45^. Our studies propose another layer of regulation: we hypothesize that TDP-43 affects transcription through misregulation of LEC and SEC activities, contributing to degeneration. Furthermore, the data showing that TDP-43 interacts with ELL2 (Fig. 6c) suggests a mechanism, which TDP-43 may promote the activities of LEC and SEC at target genes, through interactions of the TDP-43 with ELL2 (Fig. 7).

Our data highlight elements of specificity in TDP-43-associated toxicity. First, we show that only selected targets of LEC and SEC are elevated in expression in the fly upon TDP-43 expression. The selectivity of targets can be defined by accessory factors associated with the LEC and SEC: in human cells, a mediator subunit MED26 has been shown to interact through EAF1 and EAF2 and promote the expression of select target genes, including a subset of snRNAs and Hsp70^46,47^. Other factors like PAF1 and integrator, interacting with SEC^48,49^, might also contribute to target specificity.

Our data showing that TDP-43 binds to *Drosophila* polytene chromosomes with selectivity (Fig. 3a, b), and that TDP-43 interacts with ELL2 (Fig. 6c), suggest specificity could also be defined by TDP-43. In HIV infection and leukemia, the sequence-specific DNA-binding activators, HIV-1 Tat protein and MLL, respectively, direct SEC to specific target genes for their abnormal expression^17,50,51^. Thus, it is possible that TDP-43 serves as a sequence-specific DNA- or RNA-binding factor and therefore defines target specificity in associated diseases. A second layer of specificity is that, upon increased U12 snRNA levels, among 18 target genes with a U12-type intron, only six become misregulated with TDP-43. The detailed mechanisms by which specificity is established is an intriguing question that remains to be addressed.

In models of the motor neuron disease spinal muscular atrophy (SMA), expression levels and splicing of several genes containing a U12-type intron are decreased^32^. Our data show that the activity of the minor spliceosome is affected in an opposite direction in TDP-43 expressing animals, highlighting the importance of a balanced level of the minor spliceosome in maintaining normal motor neuron function. Products of six minor spliceosome-regulated genes were increased with TDP-43, with three genes potentially regulated through transcription and three genes likely through misregulation of splicing (see Fig. 2e and Supplementary Fig. 2f, g); among these, expression of three genes (*CG33108*, *CG16941*, and *CG11839*) are decreased in a fly model of SMA^32^. This finding indicates misregulation of common target genes in different motor neuron disease models. Misregulation of orthologues of some of these genes have been reported in mammalian models of ALS and FTD or in human patients: the splicing of a U12-type intron in *C19orf54*, the human orthologue of *CG33108*, is affected in an ALS transgenic mouse model expressing human FUS, which is encoded by a gene that, when mutated, can cause ALS and rare cases of FTD^52^. Another gene, *SF3A1*, the human orthologue of *CG16941*, shows altered poly-A usage in ALS patients bearing the *C9orf72* GGGGCC hexanucleotide repeat expansion^22^, the most common known genetic cause of ALS and FTD^22,53,54^. *SF3A1* is also implicated as a novel risk factor in FTD by gene co-expression network analysis^55^. Our findings, together with others, indicate that these genes may be critical in TDP-43-associated disorders.

We report that the stress-induced repetitive RNA Sat III is increased in TDP-43-associated disease, which suggests that the elevation may contribute to neuronal loss and degeneration. We used both a HEK293 cell disease model and human patient samples to verify findings in different systems. Stress genes maintain proteostasis and promote cell survival. However, the stress response can be diverse depending on the circumstances^56,57^. The induction of Sat III by TDP-43 is significant although mild (~4-fold; see Fig. 5), leading us to consider that expression of TDP-43 may be akin to a chronic stress that triggers distinct pathways from those induced by an acute heat shock response (whereby Sat III is induced many thousand-fold (see Supplementary Fig. 4)). In support of this idea, although Sat III is required for cell survival after heat shock, forced expression of Sat III triggers cell death and rapid cellular senescence^38,39^. Elevated Sat III transcripts have been noted in senescent cells and fibroblasts from patients with the premature aging disease Hutchinson–Gilford progeria syndrome^58,59^. Furthermore, normalization of elevated Sat III largely rescues the mitotic dysregulation and senescence phenotype of SIRT6-depleted cells^39^, indicating that elevated Sat III contributes to age-related cellular abnormalities. These studies support our findings and model that elevated Sat III is detrimental and may contribute to TDP-43-associated neuronal dysfunction. The influence of ELL proteins on the induced Sat III levels by TDP-43 is an interesting question that remains to be further investigated.

Given our data in *Drosophila*, the shared component *Ell*, whose depletion rescued the elevated levels of LEC and SEC targets, may be an effective therapeutic target. Importantly, our data also showed that knockdown of *Ell* on its own did not cause deleterious effects like degeneration or compromised mobility, further supporting that *Ell* may be a promising therapeutic candidate (see Supplementary Fig. 1b, f). Domain analysis of human orthologues of Ell, ELL, and ELL2, identified the N-terminal 150 amino acids as critical for elongation function^60,61^. Given that the ELL family is also reported to display additional activities, such as serving as an E3 ubiquitin ligase for c-Myc degradation^62^ and inhibiting P53 function^62^, a strategy to apply molecules targeting domains specific for elongation activity, or even targeting the interaction between ELL proteins and the assembly components of LEC and SEC, ICE1 and AFF4 (human orthologue of Lilli), respectively, may be promising. Recent research resolving the structure of the AFF4 and ELL2 binding interface reveals a cavity that is a potential binding site for small molecules to interrupt SEC activity^63^.

The therapeutic potential of ELL family proteins may not be limited to TDP-43-associated toxicity. Our findings indicate that knockdown of *Ell* protects against not only TDP-43 but also GGGGCC hexanucleotide repeat toxicity (Supplementary Fig. 9), which is also a major disease locus for ALS and FTD. These data suggest that shared targets of these two toxic insults and of ELL family proteins might be central to degeneration associated with these mechanisms.

## Methods

### *Drosophila melanogaster*

Flies were raised at 25 °C. Transgenic flies with TDP-43-YFP and recombinant fly lines gmr > TDP-43 and elavGS > TDP-43 are described^15,16^. The recombinant fly line daGS > TDP-43 was generated with the fly line *daughterless-GeneSwitch* (*dsGS*), a generous gift from Dr. Veronique Monnier^64^. Two *Ice1* RNAi fly lines were generated with constructs (SH09112.N and SH09113.N) provided by DRSC/TRiP center. Fly lines used are listed in Supplementary Table 3.

### *Drosophila* external eye and internal eye imaging

Adult female flies (1–2 days) were used. For external eyes, flies were anesthetized with ether and imaged. For retinal tissue imaging, fly heads were embedded in paraffin blocks and sectioned for images of endogenous autofluorescence. Images of retina sections were analyzed by ImageJ to measure the thickness of retina tissue in the middle for quantification.

### RU486 induction in adult fly

Adult male flies (0–1 days) were collected and aged in vials containing fly media added with or without RU486 as indicated (4 mg/ml or 8 mg/ml, 100 μl) to induce gene and RNAi expression at 25 °C for indicated days. In all, 100% ethanol (EtOH) was used as vehicle.

### Adult fly climbing assay

Adult male flies were collected for RU486 induction. Assays were conducted between 10 and 11 a.m. Plastic vials (height = 9.5 cm) were used for the assay. Flies were transferred into plastic vials at the density of 25 flies per vial 20 min before the assay. For the assay, flies were tapped to the bottom of the vial, and the number of flies climbing above 5.5 cm was counted after 15 s (three repeats). For each trial, a cohort of 100 flies was assessed for each genotype over 21 days.

### Fly lifespan assay

Male flies were collected 0–1 days after eclosion and transferred into vials containing fly media with or without RU486 at a density of 20 flies per vial. Flies were transferred to fresh media every other day and the numbers of dead flies were scored. For each group, 200 flies were used in each experiment.

### Western blotting

Adult male fly heads (1–2 days unless otherwise noted) were homogenized in Laemmli sample buffer (Bio-Rad) with βME, boiled and centrifuged to remove debris. NuPAGE 4–12% Bis-Tris gel (ThermoFisher) was used to run the samples. Proteins were transferred to nitrocellulose membrane by the iBlot blotting system (ThermoFisher). For human cell samples, cells (~9.6 × 10^5^) were lysed in RIPA buffer (Cell Signaling; #9806) supplemented with phenylmethylsulfonyl fluoride (PMSF) and protease inhibitor cocktail (Roche; #1183670001). Extracts were sonicated for 6 min using using QSonica (Newtown, CT) water bath sonicator (amplitude = 100, 30-s on, 30-s off). After centrifugation, the concentrations of the protein samples were measured by Pierce BCA kit (Thermo; #23225). The same amount of protein was prepared for each sample with NuPAGE LDS sample buffer (Invitrogen; #NP0007), boiled at 95 °C, 5 min and run on NuPAGE 4–12% Bis-Tris gel (ThermoFisher). Proteins were transferred to polyvinylidene difluoride (PVDF) membrane by the XCell SureLock system (Invitrogen). Primary antibodies used were anti-TDP-43 rabbit polyclonal antibody (1:5000; Proteintech; #10782-2-AP), anti-β-gal mouse monoclonal antibody (1:2000; Promega; #Z378A), anti-α-tubulin rabbit polyclonal antibody conjugated with horseradish peroxidase (HRP) (1:1000; Cell Signaling; #9099), anti-ELL rabbit polyclonal antibody (1:1000; Proteintech; #51044-1-AP), anti-ELL2 rabbit polyclonal antibody (1:1000; Bethyl; #A302-505A), anti-ELL2 mouse monoclonal for co-IP immunoblots (1:1000; Santa Cruz; #sc-515276), and anti-GAPDH mouse monoclonal (1:10,000; AbD Seotec; #4699-9555). The secondary antibodies used were goat anti-rabbit IgG-HRP (1:5000; Milipore, #AP307P) and goat anti-mouse (1:5000; Jackson ImmunoResearch; #115-035-146). All blocking and antibody incubations were done in 5% milk in phosphate-buffered saline (PBS) overnight (O/N) at 4 °C for primary and 1 h at room temperature (RT) for secondary. Signals were developed by ECL plus (ThermoFisher) or ECL prime (GE healthcare) western blotting reagents. The images were scanned by Fujifilm LAS-3000 imager (Fujifilm) or Amersham Imager 600 (GE healthcare), and quantification was performed using ImageJ. The uncropped blots in the main figures are provided in Supplementary Fig. 7.

### Small RNA northern blotting

Total RNAs were extracted from fly heads using Trizol reagent (ThermoFisher; #15596026), following the manufacturer’s protocol. RNA quality was checked by gel, and 0.6–3 μg of total RNA was loaded in 15% TBE-urea gel (ThermoFisher; #EC68852BOX). RNAs were then transferred onto nylon membrane (GE HealthCare; #RPN303B) and cross-linked by ultraviolet (UV). The membrane was then prehybridized by UltraHyb Oligo Hybridization Buffer (ThermoFisher; #AM8663) and then hybridized with P^32^-labeled probes overnight at 50 °C. To make the probes, DNA oligos were annealed to obtain the template for RNA probes, which were synthesized by in vitro transcription using MAXIscript T7 kit (ThermoFisher; #AM1312), supplemented with P^32^-α-UTP. Signals were detected by GE Amersham Typhoon 9410 Imager and analyzed by ImageJ for quantification. Probes used are listed in Supplementary Table 4. The same blots were used to probe 2 different snRNAs: either U1 and U11; U2 and U5; U4 and U7 or U4atac and U12. The uncropped scans of the blots are shown in Supplementary Fig. 8.

### *Drosophila* polytene immunohistochemistry

To obtain larvae in the approximately same developmental stage, fly food was mixed with 0.05% bromophenol blue, and light blue wandering larvae were selected for experiments. Salivary glands were dissected in PBST (0.05% Tween20) and fixed with 45% acetic acid and 2% paraformaldehyde for 1 min and transferred into a drop of 45% acetic acid on a sigma-coated coverslip. The salivary glands were then squashed onto Fisherbrand Superfrost Plus Microscope slides (Fisher Scientific), and liquid nitrogen was used to freeze the slides. Slides were blocked at room temperature (RT) for 1 h, and incubated with anti-GFP mouse monoclonal antibody (1:500; Takara; #632380), anti-Lilli rabbit polyclonal antibody (1:500; a generous gift from Dr. Ali Shilatifard) and anti-Ell rabbit polyclonal antibody (1:500; a generous gift from Dr. Ali Shilatifard) overnight at 4 °C. The secondary antibodies were goat anti-mouse IgG Alexa Fluor488 (1:500; ThermoFisher; #A-11001) and goat anti-rabbit IgG Alexa Fluor488 (1:500; ThermoFisher; #A-11037). All blocking and antibody incubations were done in 3% bovine serum albumin in TBST (0.05% Tween 20). 4,6-Diamidino-2-phenylindole (DAPI; ThermoFisher; #D3571) was used for nucleic acid staining. The spreads were mounted using Dako fluorescence mounting medium (Dako; #S3023) and imaged on a Leica DM6000 CS confocal microscope. For heat shock treatment, larvae were incubated for 5 min at 37 °C.

### Reverse transcription quantitative PCR

Total RNA from larvae, whole fly, fly heads, human cultured cells or human tissue was extracted using Trizol reagent (ThermoFisher; #15596026), following the manufacturer’s protocol. DNA was removed by Turbo DNA-free kit (ThermoFisher; #AM1907) for fly samples, RNeasy micro kit (QIAGEN; #74004) for human cultured cells or DNase I (ThermoFisher; #18068015) for human tissue samples. RNA quality was checked by gel or Agilent 2100 Bioanalyzer system (Agilent). Reverse transcription was performed with random hexamers using High-Capacity cDNA Reverse Transcription kit (ThermoFisher; #4368814) for samples from fly tissues and human cultured cells or SuperScript III Reverse Transcriptase (ThermoFisher; #18080093) for human tissue samples. Fast SYBR green master mix (ThermoFisher; #4385614) was used for qPCR performed by ViiA 7 Real-Time PCR system (ThermoFisher). Primers used for qPCR are listed in Supplementary Table 5, 6. Sequences of primers for *CG6323*, *CG8408*, *CG16941*, *CG11839*, *CG7892*, *CG13431*, *CG33108*, *CG16941* U12int, and *CG11839* U12int are as previously described^32^.

### ChIP-seq data analysis

Raw fastq data from GSM345570 and GSM345568 (input control and Lilli ChIP-seq, respectively) were used to call Lilli peaks^19^. Raw fastq data from GSM2224492 and GSM2224493 (input control replicates), GSM2224501 GSM2224502, GSM2224503, and GSM2224504 (TBPH ChIP-seq) were used to call narrow TBPH peaks^11^. Narrow peaks were called using the mosaics R package^65^. Read trimming, alignment, and peak calling steps were performed in R using a published pipeline^66^. Software packages used were mosaics v2.12.0, Rbowtie v1.14.0, dada2 v1.2.2, quasR v 1.14.0, TxDb.Dmelanogaster.UCSC.dm6.ensGene v3.3.0, GenomicRanges v1.26.4, GenomicFeatures v1.26.4, BSgenome v1.42.0, biomaRt v2.30.0, AnnotationDbi v1.36.2, deeptools v3.1.0, python v2.7.10, Integrative Genomics Viewer v2.3.93, and R v3.3.2. Mosaics peaks were called using the default parameters, with the exceptions of analysis type IO, false discovery rate (FDR) = 0.05. Only regions with a called mosaics narrow peak in all biological replicates, when compared to both controls, were counted as a peak. If these peaks lay within 250 bp of a transcription start site, the corresponding gene was considered to have a peak.

### Human cell culture

The doxycycline-inducible (TetON) HEK293 cell lines, TetOn-GFP (clone#9.3), and TetOn-GFP-TDP-43 (clone#12.5) generated using a subclone of HEK293 cells (QBI-293) are generous gifts from Dr. Virginia Lee’s laboratory. Cells were grown in Dulbecco’s modified Eagle’s medium (DMEM) with l-glutamine, glucose, and sodium pyruvate (Corning; #MT10013CV), supplemented with 10% Tet system-approved fetal bovine serum, 1% penicillin/streptomycin. Media were further added 400 μg/ml G418 and 1 μg/ml puromycin. Cells were cultured at 37 °C and 5% CO_2_ and routinely sub-cultured at 1:10 ratio every 7 days. To induce expression, 1000 ng/ml doxycycline was added for 6 days. For heat shock treatment, cells were subjected to heat stress at 46 °C followed by 6-h recovery. Cells were checked for appropriate GFP-tagged protein expression.

### Human frontal cortical tissue

Human postmortem brain samples were obtained from the University of Pennsylvania Center for Neurodegenerative Disease Brain Bank. All relevant ethical regulations were compiled, and informed consent from next of kin was obtained for all cases. These comprised samples from clinically normal individuals (*n* = 6), as well as individuals with FTLD-TDP (*n* = 13). The region sampled was midfrontal cortex (BA9), and all disease cases were previously reported and confirmed to have TDP-43 pathology^40,41^. DNA was extracted from all cases and screened for mutations in the two most common genes associated with FTLD-TDP, *GRN*, and *C9orf72*. Briefly, the coding regions of *GRN* were bi-directionally sequenced by Sanger sequencing using flanking primers to each exon as previously described^67^. Sequence analysis was done with Mutation Surveyor (SoftGenetics, State College, PA). Analysis for hexanucleotide repeat expansions in *C9orf72* was performed using a modified repeat-primed PCR method^68^. Analysis of the *valosin containing protein*(*VCP*) gene was performed by targeted Sanger sequencing of the relevant exon in a case with known family history of a *VCP* mutation^69^.

### Co-immunoprecipitations

Endogenous co-IP was performed by using the nuclear extract of HEK293 TetOn-GFP (clone#9.3) cells without doxycycline induction. Cells (~2.6 × 10^7^) were resuspended in hypotonic solution (20 mM Tris-HCl (pH 7.5), 20 mM NaCl, 5 mM MgCl_2_) supplemented with protease inhibitor cocktail (Roche; #1183670001) and homogenized by Dounce homogenizer. After centrifugation (3000 *g*, 4 °C, 15 min), the pellet containing nucleus was resuspended in Pierce IP lysis buffer (Thermo; #87787) supplemented with protease inhibitor cocktail (Roche; #1183670001). After centrifugation (15,000 *g*, 4 °C, 10 min), input sample was saved, and the rest of the lysate were divided to incubate at 4 °C, overnight with Dynabeads Protein G (Invitrogen; #1004D) prepared with 5 μg of anti-TDP-43 mouse monoclonal antibody- mAb 5028^70^ (a generous gift from Dr. Virginia Lee’s lab) or same amount of mouse IgG control (Santa Cruz; #sc-2025). The next day, the beads were washed with lysis buffer three times, with the third time rotating the tubes for 5 min in 4 °C. Elute the proteins by NuPAGE LDS sample buffer (Invitrogen; #NP0007) by boiling at 95 °C, 5 min. The following western blotting were performed as described above. Experiments were repeated to confirm the results.

### Statistical analysis

Graphs are represented as mean ± standard deviation (SD). The statistics used are indicated in each figure legend. Comparison between two groups were calculated using the two-tailed unpaired Student’s *t*-test. If data sets show significant variance according to variance *F*-test analysis, two-tailed unpaired Student’s *t*-test with Welch’s correction was used as indicated in the figure legend. Shapiro–Wilk normality test was used for testing normal distribution for all data sets except data sets normalized to 1. If the data were not normally distributed, two-tailed unpaired Mann–Whitney test (nonparametric test) was used as indicated in the figure legend. The differences among three groups were calculated using analysis of variance (ANOVA) followed by Tukey’s multiple comparison test. Brown–Forsythe test and Shapiro–Wilk normality test were used to test variance differences and normality, respectively, for data analyzed by one-way ANOVA. Differences with *P*-values < 0.05 were considered statistically significant. The number of sample size and biological replicates is indicated in the Methods section or figure legend. No statistical method was used to predetermine sample sizes. No sample was excluded from the analysis. No method of randomization was used. The investigators were not blinded to allocation during experiments.

### Code availability

The code used for analysis is available upon request.

## Electronic supplementary material

Supplementary Information

Description of Additional Supplementary Files

Supplementary Data 1

## Data Availability

The data that support the findings of this study are available from the corresponding author upon reasonable request.
