# Author Correction: Aberrant activation of non-coding RNA targets of transcriptional elongation complexes contributes to TDP-43 toxicity

**DOI:** 10.1038/s41467-019-08562-x

**Published:** 2019-01-29

**Authors:** Chia-Yu Chung, Amit Berson, Jason R. Kennerdell, Ashley Sartoris, Travis Unger, Sílvia Porta, Hyung-Jun Kim, Edwin R. Smith, Ali Shilatifard, Vivianna Van Deerlin, Virginia M. -Y. Lee, Alice Chen-Plotkin, Nancy M. Bonini

**Affiliations:** 1 Department of Biology, University of Pennsylvania, Philadelphia, PA 19104 USA; 2 Cell and Molecular Biology Graduate Group, Perelman School of Medicine, Philadelphia, PA 19104 USA; 3 Department of Neurology, Perelman School of Medicine, Philadelphia, PA 19104 USA; 4 Department of Pathology and Laboratory Medicine, Perelman School of Medicine, Philadelphia, PA 19104 USA; 5 Department of Biochemistry and Molecular Genetics, Feinberg School of Medicine, Northwestern University, Chicago, IL 60611 USA; 6 Present Address: Department of Neural Development and Disease, Korea Brain Research Institute (KBRI), Daegu, 41068 South Korea

Correction to: *Nature Communications*; 10.1038/s41467-018-06543-0; published online 23 Oct 2018.

The original version of this Article contained an error in the author affiliations. The affiliation of Alice Chen-Plotkin with the Department of Neurology, Perelman School of Medicine, Philadelphia, PA, 19104 USA was inadvertently omitted. This has now been corrected in both the PDF and HTML versions of the Article.

