# Supplementary material for: Aberrant activation of non-coding RNA targets of transcriptional elongation complexes contributes to TDP-43 toxicity
Source: Nat Commun. 2018 Oct 23;9:4406. doi: 10.1038/s41467-018-06543-0 (PMC6199344; doi:10.1038/s41467-018-06543-0)
Supplement: Supplementary file 1 — Supplementary Information [file 41467_2018_6543_MOESM1_ESM.pdf]

Supplementary Information for:

Aberrant activation of non-coding RNA targets of elongation complexes LEC and SEC contributes to TDP-43 toxicity

Chia-Yu Chung[1,2], Amit Berson[1], Jason R. Kennerdell[1], Ashley Sartoris[1], Travis Unger[3], Sílvia Porta[4], Hyung-Jun Kim[1,†], Edwin R. Smith[5], Ali Shilatifard[5], Vivianna Van Deerlin[4], Virginia M-Y Lee[4], Alice Chen-Plotkin[2], and Nancy M. Bonini[1*]

[1]Department of Biology, University of Pennsylvania, Philadelphia, PA, 19104, USA
[2]Cell and Molecular Biology Graduate Group, Perelman School of Medicine, Philadelphia, PA, 19104, USA
[3]Department of Neurology, Perelman School of Medicine, Philadelphia, PA, 19104, USA
[4]Department of Pathology and Laboratory Medicine, Perelman School of Medicine, Philadelphia, PA, 19104, USA
[5]Department of Biochemistry and Molecular Genetics, Feinberg School of Medicine, Northwestern University, Chicago, IL, 60611, USA

*Corresponding author: nbonini@sas.upenn.edu
†Current address: Department of Neural Development and Disease, Korea Brain Research Institute (KBRI), Daegu, South Korea, 41068

Supplementary Tables 1-5
Supplementary Figures 1-6

Description of additional supplementary file

File name: Supplementary Data 1
Legend: The lists of genes bound by TBPH, Lilli or both, derived from [1, 2]

Supplementary Table 1. Quantification of co-localization of TDP-43-YFP and Lilli on 16 sites in 4 polytenes, related to Figure 3.

Chromosome	~Cytological bands	Polytene 1	Polytene 2	Polytene 3	Polytene 4
X	2E	+	+	+	+
	3F	+	?	+	+
	15C	+	+	+	+
	17C	+	+	+	+
	18B	+	+	+	+
2L					
2R	43E	+	+	+	+
	48A	+	+	+	+
	60A/B	+	+	+	+
3L	61C	+	+	+	+
	62A	+	+	?	+
	74 EF	+ (weak)	+	+	+
	75CD	+ (weak)	+	+ (weak)	+
3R	82D	+	+	+	+
	93D	+	+	+	+
	95F	+	+	+	+
	100C	+	+	+	+

(+) co-localization, (?) unable to define due to pattern of chromosome spreading.

Supplementary Table 2. Quantification of co-localization of TDP-43-YFP and Lilli on 6 major heat shock loci in 15 polytenes, related to Figure 3.

Major heat shock loci	63B	67B	93D	95D	87A	87C
Polytene 1	+ (weak)	+ (weak)	+	-	-	-
Polytene 2	-	+	+	-	-	-
Polytene 3	+ (weak)	-	+	-	-	-
Polytene 4	+ (very week)	+ (weak)	+	-	-	-
Polytene 5	+ (weak)	-	+	-	-	-
Polytene 6	+ (weak)	-	+	-	-	-
Polytene 7	+ (weak)	-	+	-	-	-
Polytene 8	-	-	+ (weak)	-	-	-
Polytene 9	-	-	+	-	-	-
Polytene 10	+	-	+	-	-	-
Polytene 11	+ (very weak)	-	+	-	-	-
Polytene 12	+ (very weak)	-	+	-	-	-
Polytene 13	-	-	+	-	-	-
Polytene 14	-	-	+	-	-	-
Polytene 15	-	-	+	-	-	-

(+) co-localization, (-) no co-localization.

Supplementary Table 3. Fly lines.

Fly lines	chromosome(s)	Reference/Source
w^{1118}	1	Bloomington
UAS-TDP-43-YFP	3	[3]
UAS-TDP-43	2	[3]
UAS-TDP-43	3	[3]
elavGS-GAL4	3	Bloomington
daGS-GAL4	2	[4]
da-GAL4	3	Bloomington
gmr-GAL4(YH3)	3	[3]
sgs3-GAL4	3	Bloomington
UAS-LacZ	2	Bloomington
lilli$^{17\text{-}2}$	2	Bloomington
Hsrω^{66}	3	Bloomington
UAS-Ell$^{P\{EP\}G4098}$	3	Bloomington
UAS-Ell.RNAi^{HMS00277}	3	Bloomington
Ell$^{[S\text{-}192]}$	3	Bloomington
UAS-ear.RNAi^{HMS00107}	3	Bloomington
UAS-Ice1.RNAi (SH09112.N from DRSC/TRiP)	2	This paper
UAS-Ice1.RNAi (SH09113.N from DRSC/TRiP)	3	This paper
UAS-Ice1.RNAi (13550R-2 from NIG)	2	NIG
UAS-snRNA:U12:73B.RNAi^{HMC03841}	2	Bloomington
UAS- Hsrω.RNAi^{HMC05093}	2	Bloomington
UAS-Control.RNAi^{JF01355}	3	Bloomington
UAS-$(G_4C_2)_{48}$	2	[5]
UAS-mCherry.RNAi	3	Bloomington

Bloomington is to the Bloomington Drosophila Stock Center, https://bdsc.indiana.edu/

Supplementary Table 4. Probes for small RNA Northern blot.

Name	Sequence
U1	5′-GATAATACGACTCACTATAGGGAGA-3′<br>5’-AAAAAACTGAGTTGACCTCTGCGATTATTCCTCTCCCTATAGTGAGTCGTATTATC-3′
U2	5′-GATAATACGACTCACTATAGGGAGA-3′<br>5’-AAAAAATCGGCCTTATGGCTAAGATCAAATCTCCCTATAGTGAGTCGTATTATC-3′
U4	5′-GATAATACGACTCACTATAGGGAGA-3′<br>AAAAAAGAAAACTTTAACCAATACCCCGCCTCTCCCTATAGTGAGTCGTATTATC-3′
U4atac	5′-GATAATACGACTCACTATAGGGAGA-3′<br>5’-AAAAAATCAATGAACGTCTAGTGAGGACATTTCTCCCTATAGTGAGTCGTATTATC-3′
U5	5′-GATAATACGACTCACTATAGGGAGA-3′<br>5’-AAAAAATCTGGTTTCTCTTCAATTGTCGAATTCTCCCTATAGTGAGTCGTATTATC-3′
U7	5′-GATAATACGACTCACTATAGGGAGA-3′<br>5’-AAAAAACTCTTTGAAATTTGTCTTGGTGGGATCTCCCTATAGTGAGTCGTATTATC-3′
U11	5′-GATAATACGACTCACTATAGGGAGA-3′<br>5’-AAAAAAGTTTCCGATCACGAAACTCAAGTGTCTCCCTATAGTGAGTCGTATTATC-3′
U12	5′-GATAATACGACTCACTATAGGGAGA-3′<br>5’-AAAAAAAATGAGTAAGGAAAACCAATCAGCCTCTCCCTATAGTGAGTCGTATTATC-3′
2S	5′-GATAATACGACTCACTATAGGGAGA-3′<br>5’-TGCTTGACTACATATGGTTGAGGGTTGTATCTCCCTATAGTGAGTCGTATTATC-3′

Two DNA oligonucleotides were annealed to obtain the template for each RNA probe.

Supplementary Table 5. qPCR primers for *Drosophila* genes.

Name	Forward Sequences (5'-3')	Reverse Sequences (5'-3')
Ell	AAAACAAACTCACATACAACCAAAAA	GCACTCGTTGTGGAAGTAGACA
Ear	TTCCGAAACCCAAACGAGT	CGTCTCGGTTGCGAAAGTA
Ice1	ACCACTTCCAGCCCTATGAG	GCTTCCCCACCAGGTTCT
U12	TTTGCAAGGGCACAGGTC	GCTAGCCGGACGCAAAGTAG
CG6323	TGCTCTTCTTCTACATGATCATTCTGTT	GCACTTGTTTGCGCAAATCC
CG8408	GTCGCCTACGTATTCCTGCAAACA	CCAGCGCCGAACAGAAACATATGA
CG16941	GCCGGAAGTTAGAAATATCGTTGAC	ACTTTGGATTGCCCAGCTCA
CG11839	CACCATTGGCAAAGTATGACTCCTCG	CCACCTTATTCTGCTTAGCCTGATCG
CG7892	CTTTCAAGAAATCTATGTGATAACGGAG	GGATCTGGTACAGGAACACCTT
CG13431	ATGGCCCAAATCCTTCTGGGATGA	CCAAACGTGCGAGTGCGTGATATT
CG33108	GCCTACTAGTGCCCTACGATG	TGCGTGTCCACCAGATAACCTA
CG7736	CCTCGAGGACACTATCAGCA	CCTTCATCTGCTTGACCTCATC
CG17912	CCTCCAAACCCTGGTGCT	GGGTCCGGTGTACAACTTCTT
CG32705	CGCCCATCGTAGCCATTATC	CATGTGGAAGCCGGTCAGT
CG11328	CGTTGAGCGATTTCTTCTCC	TCGAACCCGAGTAAATTTGG
CG17228	TGACGGCATGGCTCCTAC	GGGATAGCGCACCCAGAA
CG15081	AGAAGCCGAGGCTGCTAA	TGCGTGCAATGCTTTGAG
CG11984	GCGAGCCGAATCTAGTCACA	GATGCGACGCACTCCAC
CG4894	ATTCGCATTGTGGAGTGG	GTAAACAGCCAAGGCAATACAG
CG18177	GGCAAGGACGGATTCACCT	CATCGACGCGTAGTGCTTG
CG15735	GGCCTTCGATCACAACACA	GTTGCCGTTGCACTCCTT
CG3294	GAACTGCATGCCAAGAGG	GGTGTACTCCAGTTCCGTGT
CG16941 U12int	ATGGCAACTTTAGACGCGGAA	TTGTTTTCTTTTTGGCAAAGGATGC
CG11839 U12int	GTTGTTTACACTTTCTAGCCGGTGT	GCCCGAGGAGTCATACGTATAGTTA
CG33108 U12int	GCATGTAGGTCCTTTGGACTG	CAGTGTGCTGTTCACCGTTTAC
CG11328 U12int	TAACTCCCACCGCACATGA	GGCCACCACACCAGTAAGTT
CG15735 U12int	GGCCTTCGATCACAACACA	AGAAAGTGCTCGCTTTCACC
CG3294 U12int	GCGGTACGTGAACTGCAT	CAGTCGTTACCTTTGGGACA
Hsrω-all	TATCTAATGTCCGGGGTCGT	CACAATCCGCACAATCAATC
Hsrω-n	ATAGTCCCTCGGAGGAAAGG	GCGCTCACAGGAGATCAA
βTub56D	CATCCAAGCTGGTCAGTG	GCCA TGCTCATCGGAGAT
Pgk	ATCACCAGCAACCAGAGAATTG	TGCCAGGGTGTACTTGATGTT
RpS20	CCGCATCACCCTGACATCC	TGGTGATGCGAAGGGTCTTG
RpL32	CATCCGCCCAGCATACAG	CCATTTGTGCGACAGCTTAG

Supplementary Table 6. qPCR primers for human genes.

Name	Forward Sequences (5'-3')	Reverse Sequences (5'-3')
Sat III	GTGCAATCGAATGGAATCG	CCATTCCTGTACTCGGGTTG
GAPDH	CGAGATCCCTCCAAAATCAA	TTCACACCCATGACGAACAT
ACTB	TTCCTGGGCATGGAGTC	CAGGTCTTTGCGGATGTC
ELL	GTCGGAGACGCCTGACTACT	TACTCGGCATTGAAGTCGTTC
ELL2	TGGGAGCAATTCTGCAAC	ATCCAGGCCAGTCTCTTTGA

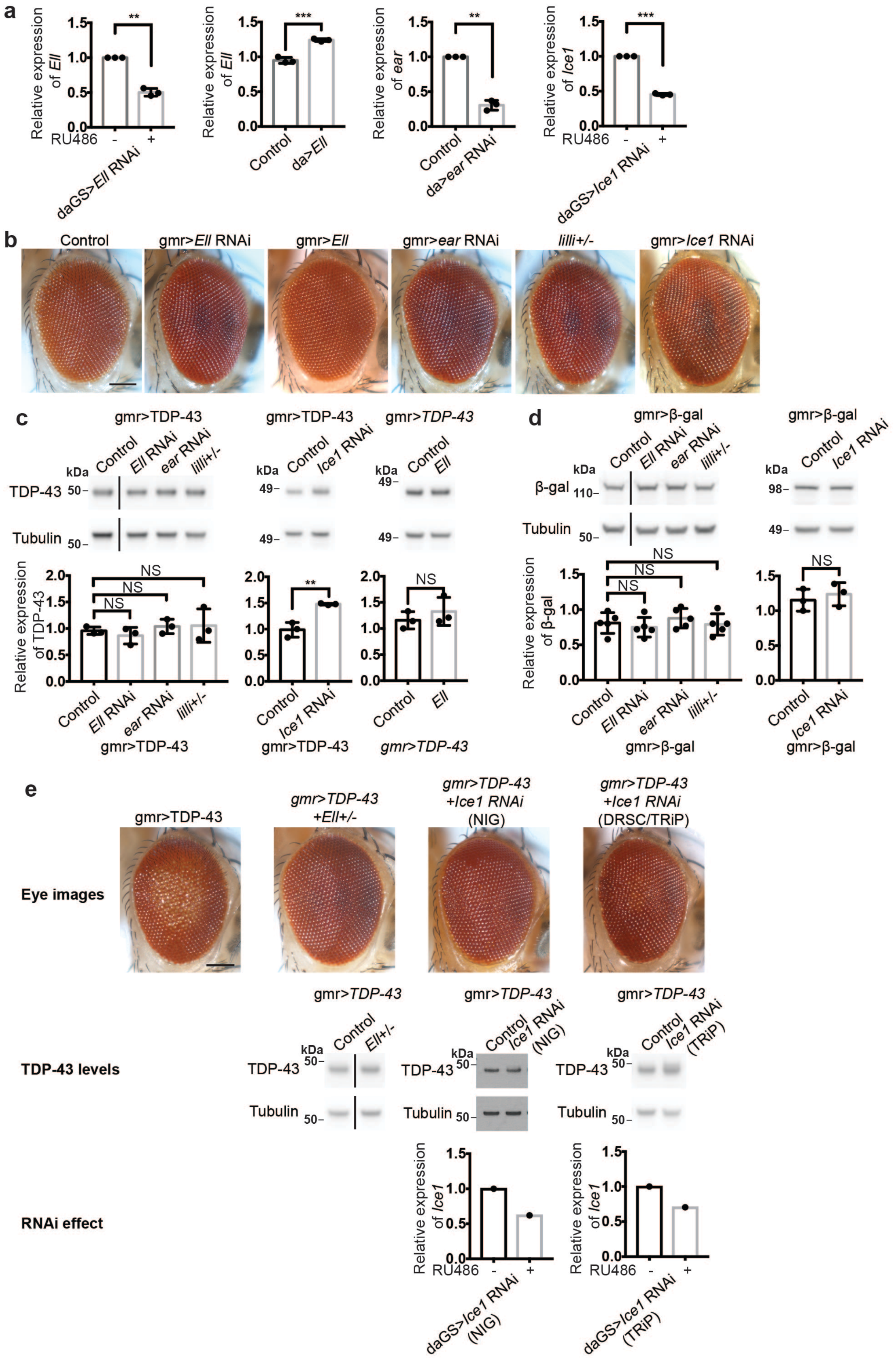
a
Relative expression of Ell
daGS>Ell RNAi
RU486 - +
**
Control
da>Ell
***
Relative expression of ear
da>ear RNAi
Relative expression of Ice1
daGS>Ice1 RNAi
b
Control
gmr>Ell RNAi
gmr>Ell
gmr>ear RNAi
lilli+/-
gmr>Ice1 RNAi
c
gmr>TDP-43
TDP-43
Tubulin
kDa
Ell RNAi
ear RNAi
Ice1 RNAi
Relative expression of TDP-43
NS
d
gmr>β-gal
β-gal
Relative expression of β-gal
e
Eye images
gmr>TDP-43 +Ell+/-
gmr>TDP-43 +Ice1 RNAi (NIG)
gmr>TDP-43 +Ice1 RNAi (DRSC/TRiP)
TDP-43 levels
RNAi effect
daGS>Ice1 RNAi (NIG)
daGS>Ice1 RNAi (TRiP)

f

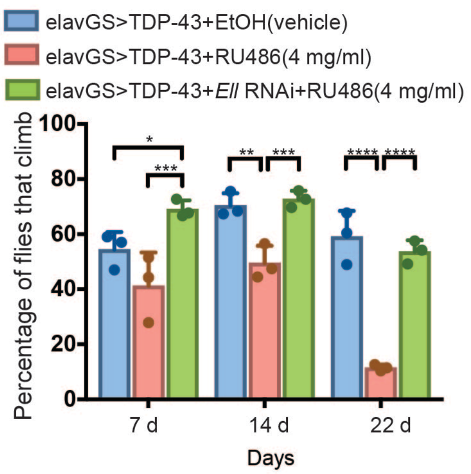
elavGS>TDP-43+EtOH(vehicle)
elavGS>TDP-43+RU486(4 mg/ml)
elavGS>TDP-43+*Ell* RNAi+RU486(4 mg/ml)
Percentage of flies that climb
100
80
60
40
20
0
*
***
**
***
****
****
7 d
14 d
22 d
Days

g

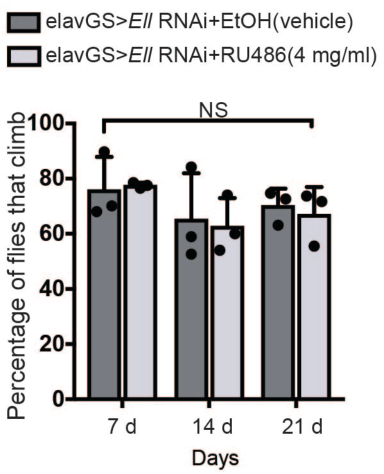
elavGS>*Ell* RNAi+EtOH(vehicle)
elavGS>*Ell* RNAi+RU486(4 mg/ml)
NS
Percentage of flies that climb
100
80
60
40
20
0
7 d
14 d
21 d
Days

h

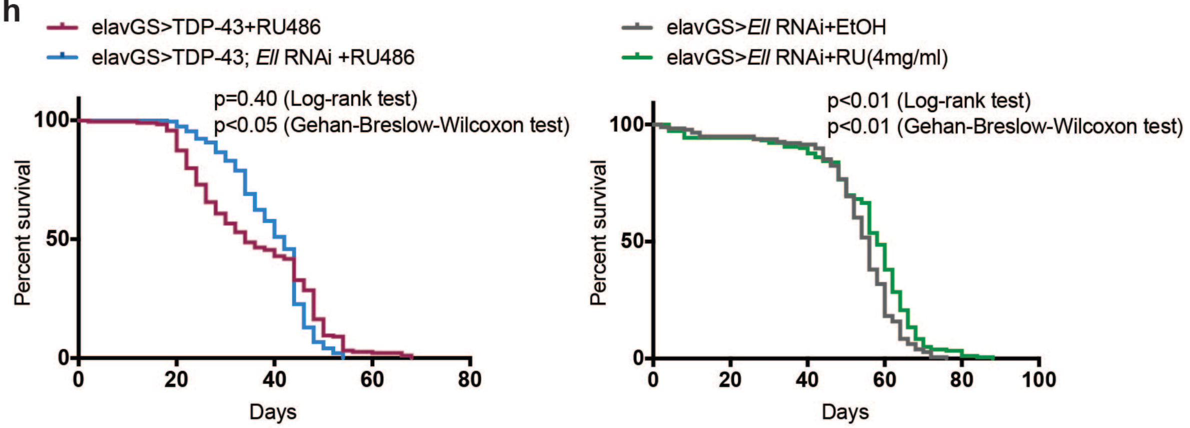
elavGS>TDP-43+RU486
elavGS>TDP-43; *Ell* RNAi +RU486
p=0.40 (Log-rank test)
p<0.05 (Gehan-Breslow-Wilcoxon test)
Percent survival
100
50
0
0
20
40
60
80
Days
elavGS>*Ell* RNAi+EtOH
elavGS>*Ell* RNAi+RU(4mg/ml)
p<0.01 (Log-rank test)
p<0.01 (Gehan-Breslow-Wilcoxon test)
Percent survival
100
50
0
0
20
40
60
80
100
Days

I

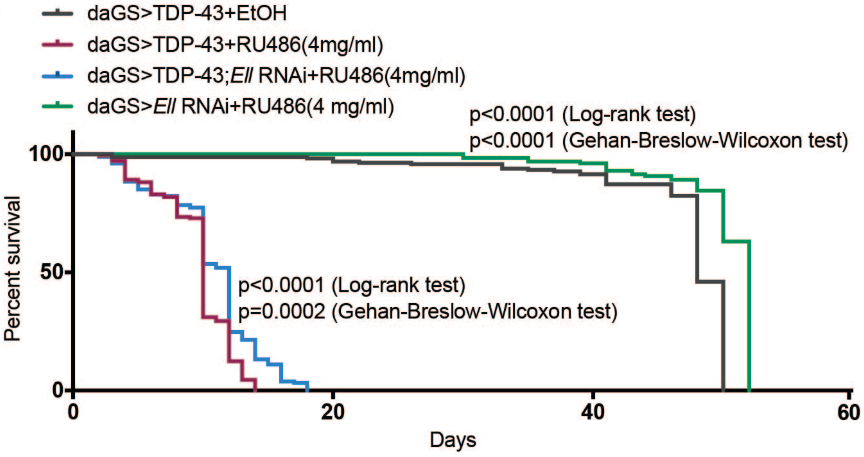
daGS>TDP-43+EtOH
daGS>TDP-43+RU486(4mg/ml)
daGS>TDP-43;*Ell* RNAi+RU486(4mg/ml)
daGS>*Ell* RNAi+RU486(4 mg/ml)
p<0.0001 (Log-rank test)
p<0.0001 (Gehan-Breslow-Wilcoxon test)
p<0.0001 (Log-rank test)
p=0.0002 (Gehan-Breslow-Wilcoxon test)
Percent survival
100
50
0
0
20
40
60
Days

Supplementary Figure 1. Downregulation of components in SEC and LEC suppresses TDP-43-caused degeneration

(**a**) RT-qPCR analysis verifies that *Ell*, *ear* and *Ice1* are knocked down by RNAi and *Ell* is elevated by the upregulation fly line. RU486 (4 mg/ml) was used to induce the expression of RNAi driven by *daGS* promoter. Whole flies were used for accessing knockdown effects of *Ell* RNAi and *Ice1* RNAi (8d RU486 induction), and upregulation effect of Ell (1d). Larvae were collect for evaluate *ear* RNAi effect. Relative mRNA levels were normalized to βTub56D mRNA. *n*=3 biological replicates. Bars represent mean (SD). (**) *P* <0.01, (***) *P* <0.001 (Two-tailed unpaired Student's *t* test with Welch's correction for RNAi effect; Two-tailed unpaired Student's *t* test for upregulation effect of *Ell*). Genotype: daGS>*Ell* RNAi is *daGS-GAL4/+; UAS-Ell.RNAi*^{HMS00277}/+, Control for *Ell* upregulation is *da-GAL4/+*, da>*Ell* is *da-GAL4/UAS-Ell*$^{P\{EP\}G4098}$, Control for *ear* RNAi is *da-GAL4/mCherry.RNAi*, da>*ear* RNAi is *da-GAL4/UAS-ear.RNAi*^{HMS00107}, daGS>*Ice1* RNAi is *daGS-GAL4/UAS-Ice1.RNAi* (SH09112.N from DRSC/TRiP).
(**b**) Reduction of components involved in SEC and/or LEC on their own does not affect eye integrity. Scale bars: 100 µm. Genotype: Control is *gmr-GAL4(YH3)/+*, gmr>*Ell* RNAi is *gmr-GAL4(YH3)/UAS-Ell.RNAi*^{HMS00277}, gmr>*Ell* is *gmr-GAL4(YH3)/UAS-Ell*^{EPG4098}, gmr>*ear* RNAi is *gmr-GAL4(YH3)/UAS-ear.RNAi*^{HMS00107}, *lilli*+/- is *lilli*$^{17-2}$/+*; gmr-GAL4(YH3)/+* and gmr>*Ice1* RNAi is *UAS-Ice1.RNAi* (SH09112.N from DRSC/TRiP)/+*; gmr-GAL4(YH3)/+*.
(**c**) The levels of TDP-43 are unaltered by *Ell* RNAi, *ear* RNAi, loss of one genomic copy of *lilli* or upregulation of *Ell*. The levels of TDP-43 are elevated by Ice1 RNAi. *n*=3 biological replicates. Bars represent mean (SD). (NS) not significant, (**) *P* <0.01 (Two-tailed unpaired Student's *t* test). Genotypes of the flies are the same as indicated in Fig. 1a, d.
(**d**) Western blot analysis of β-gal shows that the GAL4/UAS system is not altered by downregulation of components in SEC and/or LEC. *n*=3 biological replicates. Bars represent mean (SD). (NS) not significant (Two-tailed unpaired Student's *t* test). Genotypes: gmr>β-gal; Control is *gmr-GAL4, UAS-β-gal/+,* gmr>β-gal+*Ell* RNAi is *gmr-GAL4, UAS-β-gal/+; UAS-Ell.RNAi*^{HMS00277}/+*,* gmr> β-gal+*ear* RNAi is *gmr-GAL4, UAS-β-gal/+; UAS-ear.RNAi*^{HMS00107}/+*,* gmr> β-gal+*lilli+/-* is *gmr-GAL4, UAS-β-gal/lilli*$^{17-2}$*,* gmr> β-gal+*Ice1* RNAi is *gmr-GAL4, UAS-β-gal/UAS-Ice1.RNAi* (SH09112.N from DRSC/TRiP).
(**e**) Depletion of Ell and Ice1 by additional fly lines show the suppression effect on TDP-43-mediated eye degeneration. The levels of TDP-43 and RNAi effect were confirmed by western blotting and RT-qPCR. For RNAi effect confirmation, RU486 (4 mg/ml) was used to induce the expression of RNAi driven by *daGS* promoter for 8 days. Genotypes: gmr>TDP-43 is *UAS-TDP-43/+; gmr-GAL4(YH3)/+*. gmr>TDP-43+*Ell+/-* is *UAS-TDP-43/+; gmr-GAL4(YH3)/Ell*$^{[S-192]}$. gmr>TDP-43+*Ice1 RNAi(NIG)* is *UAS-TDP-43/UAS-Ice1.RNAi* (13550R-2 from NIG)*; gmr-GAL4(YH3)/+*. gmr>TDP-43+*Ice1* RNAi(TRiP) is *UAS-TDP-43/+; gmr-GAL4(YH3)/UAS-Ice1.RNAi* (SH09113.N from DRSC/TRiP). daGS>*Ice1* RNAi(NIG) is *daGS-GAL4/UAS-Ice1.RNAi* (13550R-2 from NIG) and daGS>*Ice1* RNAi(TRiP) is *daGS-GAL4/+; UAS-Ice1.RNAi* (SH09113.N from DRSC/TRiP)/+.
(**f**) TDP-43 expression in the adult neurons by *elavGS* causes climbing defects, which can be rescued by knockdown of *Ell*. Genotypes are as indicated in Fig. 1f.
(**g**) Knockdown of *Ell* on its own in neurons causes no climbing defects. Genotypes: elavGS>*Ell* RNAi is *elavGS-GAL4, UAS-Ell.RNAi* ^{HMS00277}/+.

For (**f**) and (**g**), RU486 (4 mg/ml) was used to induce the expression of TDP-43 and *Ell* RNAi. EtOH was used as vehicle. 100 flies were measured 3 times for each genotype at different time points. Bars represent mean (SD). (*) *P* <0.05, (**) *P* <0.01, (***) *P* <0.001, (****) *P*<0.0001, (NS) not significant (Two-way ANOVA followed by Tukey's multiple comparison test). Significant differences are only indicated within the same time point.
(**h**) (**I**) Lifespan analysis of flies expressing TDP-43 and/or *Ell* RNAi by neuronal driver *elavGS* (**h**) or ubiquitous driver *daGS* (**I**) show that *Ell* depletion mildly prolongs the lifespan on its own and mildly extends the shortened lifespan mediated by TDP-43 expression. Genotypes are as indicated in Fig. 1f and Fig. 2a. n=200 flies per group. *P* values are indicated in the figure (Log-rank test and Gehan-Breslow-Wilcoxon test).

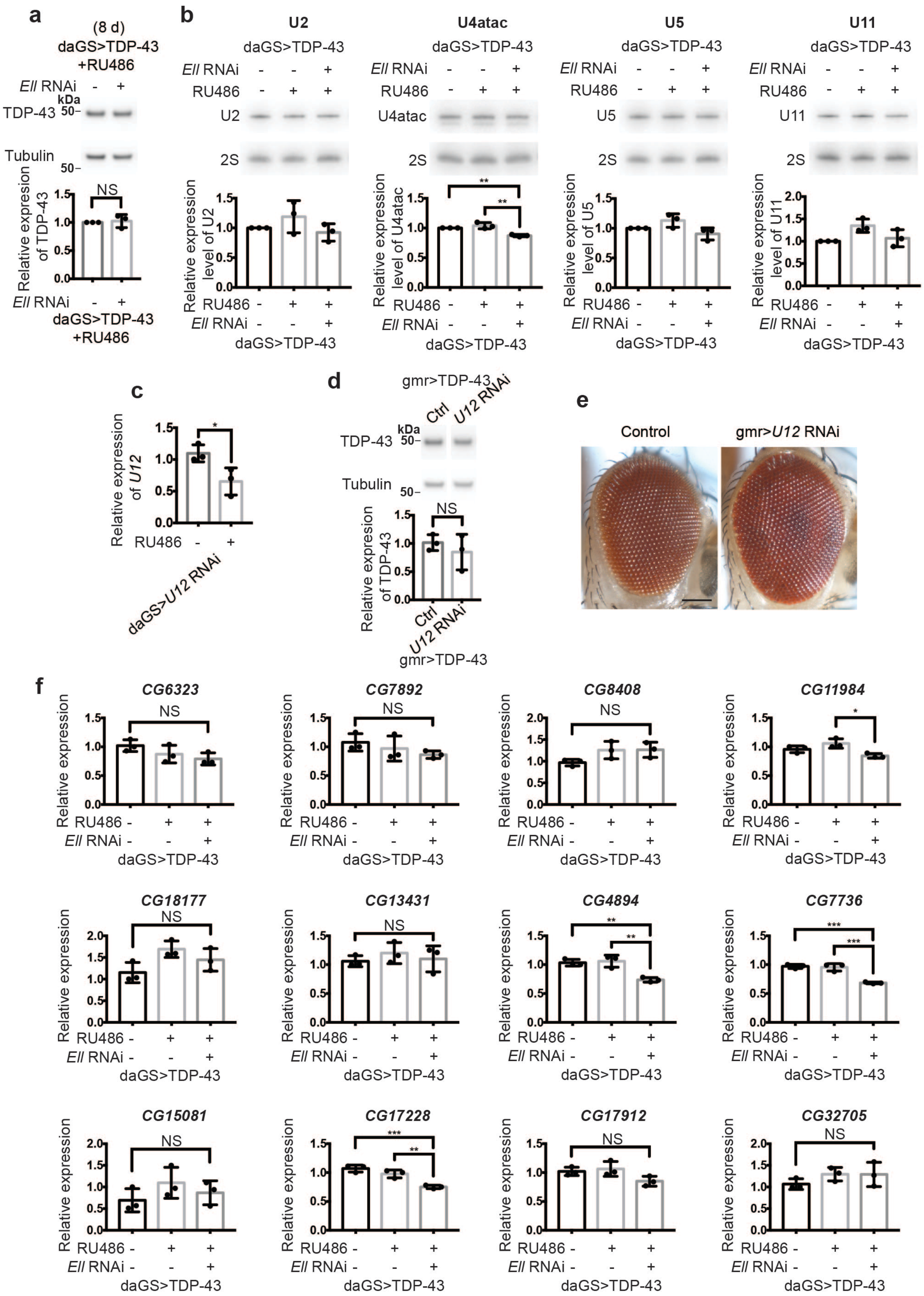
a
(8 d)
daGS>TDP-43
+RU486
Ell RNAi - +
kDa
TDP-43 50
Tubulin
50
NS
Relative expresion of TDP-43
Ell RNAi - +
daGS>TDP-43
+RU486
b
U2
U4atac
U5
U11
daGS>TDP-43
Ell RNAi - - +
RU486 - + +
2S
Relative expression level of U2
Relative expression level of U4atac
Relative expression level of U5
Relative expression level of U11
**
NS
c
Relative expression of U12
*
RU486 - +
daGS>U12 RNAi
d
gmr>TDP-43
Ctrl
U12 RNAi
TDP-43
Tubulin
Relative expresion of TDP-43
gmr>TDP-43
e
Control
gmr>U12 RNAi
f
CG6323
CG7892
CG8408
CG11984
CG18177
CG13431
CG4894
CG7736
CG15081
CG17228
CG17912
CG32705
Relative expression
RU486 - + +
Ell RNAi - - +
daGS>TDP-43
***

g

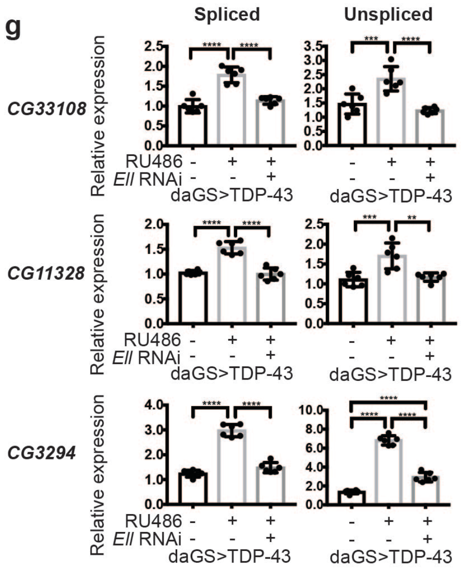

Supplementary Figure 2. Expression of TDP-43 affects the levels of selected snRNAs and U12 intron-containing genes in fly heads

(**a**) Western blot analysis shows that downregulation of *Ell* does not alter TDP-43 protein levels driven by ubiquitous drug-inducible diver *daGS* in fly heads. Genotypes of the flies and RU486 treatment are the same as indicated in Fig. 2a, e.
(**b**) Northern blot analysis of Pol II-transcribed snRNAs shows that U2, U4atac, U5 and U11 are not significantly affected by TDP-43 expression in fly heads. 2S rRNA was the internal control. Genotypes of the flies and RU486 treatment are the same as indicated in Fig. 2a, e.
(**c**) RT-qPCR analysis verifies that *U12* is knocked down by RNAi. RU486 (4 mg/ml) was used to induce the expression of RNAi for 8d, and whole flies were used. Relative mRNA levels were normalized to RpL32 mRNA. Genotype: daGS>*U12* RNAi is *daGS-GAL4/UAS-snRNA:U12:73B.RNAi*^{HMC03841}.
(**d**) Western blot analysis confirms that the levels of TDP-43 protein are not altered by *U12* RNAi. Genotypes of the flies are the same as indicated in Fig. 2b.
(**e**) Knockdown of *U12* on its own does not affect eye integrity. Scale bars: 100 μm. Genotype: Control is *gmr-GAL4(YH3)/+* and gmr>*U12* RNAi is *UAS- snRNA:U12:73B.RNAi*^{HMC03841}*/+; gmr-GAL4(YH3)/+*.
(**f**) RT-qPCR analysis of U12 intron-containing genes shows that 12 out of 18 genes are not altered by TDP-43 expression in fly heads. Relative mRNA levels were normalized to RpL32 mRNA. Genotypes of the flies and RU486 treatment are the same as indicated in Fig. 2a, e.
For (**a**-**d**) and (**f**), *n*=3 biological replicates. Bars represent mean (SD). (*) *P* <0.05, (**) *P* <0.01, (***) *P* <0.001, (NS) not significant (Two-tailed unpaired Student *t* test for comparisons between 2 groups; One-way ANOVA followed by Tukey's multiple comparison test for comparisons among 3 groups).
(**g**) RT-qPCR analysis of 18 genes regulated by U12-dependent spliceosome shows that both the spliced and unspliced products of *CG33108*, *CG11328*, *CG3294* are up-regulated by TDP-43 expression driven by *daGS* in fly heads, and downregulation of *Ell* rescues the increased levels. RU486 (4 mg/ml) was used to induce expression for 8d. mRNA levels were normalized to RpL32 mRNA. Statistical analysis and genotypes are as indicated in Fig. 2a, e.

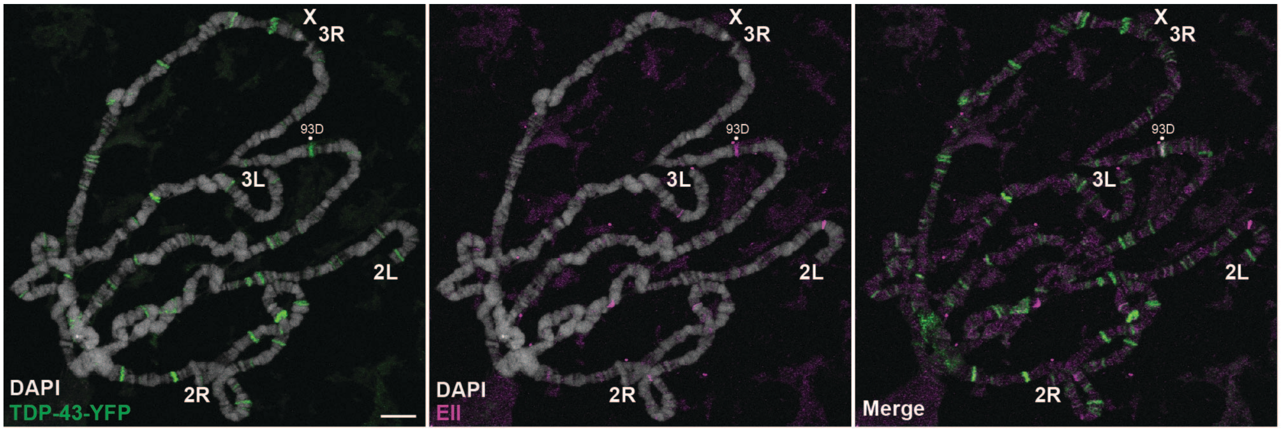

Supplementary Figure 3. TDP-43 and Ell co-localize at the 93D locus on polytene chromosomes

Immunostainings of DAPI (white), TDP-43-YFP (green) and Ell (magenta) show partial overlap and co-localization of TDP-43-YFP and Ell on *Hsrω* locus in salivary glands expressing TDP-43-YFP. Scale bars: 10 μm. Genotype is as indicated in Fig. 3a, b.

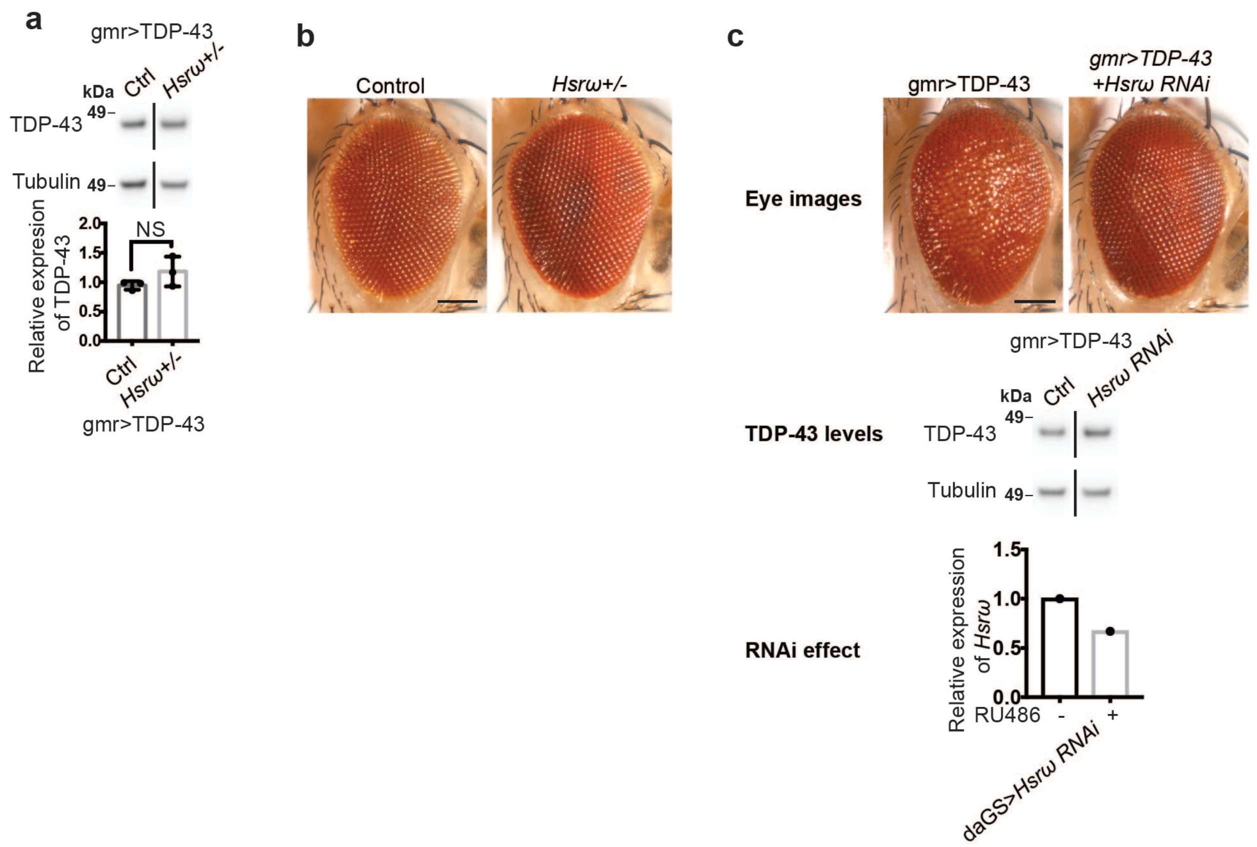

Supplementary Figure 4. Controls for the specificity of Hsrω on TDP-43 toxicity

(**a**) Western blot analysis confirms that the levels of TDP-43 are not altered by downregulation of *Hsrω*. *n*=3 biological replicates. Bars represent mean (SD). (NS) not significant (Two-tailed unpaired Student *t* test). Genotypes are as indicated in Fig. 4b.
(**b**) Loss of one copy of *Hsrω* does not disrupt eye integrity. Scale bars: 100 μm. Genotype: Control is *gmr-GAL4(YH3)/+* and *Hsrω*+/- is *gmr-GAL4(YH3)/Hsrω^{66}*.
(**c**) Depletion of *Hsrω* by an RNAi fly line suppresses the eye degeneration caused by TDP-43 expression. The levels of TDP-43 and RNAi effect were confirmed by western blotting and RT-qPCR. For RNAi effect confirmation, RU486 (4 mg/ml) was used to induce the expression of RNAi driven by *daGS* promoter for 8 days, and whole flies were used. Genotypes: gmr>TDP-43 is *UAS-TDP-43/+; gmr-GAL4(YH3)/+*. gmr>TDP-43+*Hsrω* RNAi is *UAS-TDP-43/UAS-Hsrω.RNAi^{HMC05093}; gmr-GAL4(YH3)/+.* daGS>*Hsrω* RNAi is *daGS-GAL4/UAS-Hsrω.RNAi^{HMC05093}.*

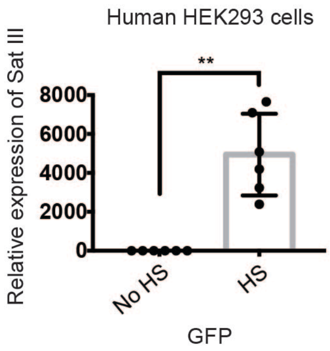

Supplementary Figure 5. Sat III qPCR primers detect a large-fold induction by heat stress

RT-qPCR analysis shows that the levels of Sat III are dramatically induced by heat stress in HEK293 cells expressing GFP after 6d induction. Relative RNA levels were normalized to *GAPDH* and *ACTB* mRNAs (geometric mean). n=6 replicates. Bars represent mean (SD). (**) *P<0.01* (Two-tailed unpaired Student's *t* test with Welch's correction).

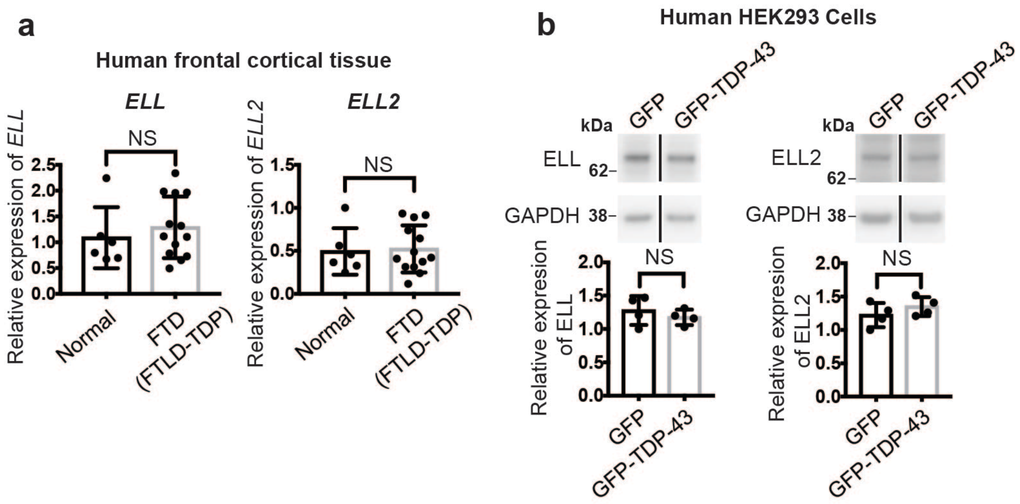

Supplementary Figure 6. RNA levels and protein levels of ELL and ELL2 are not altered in patient samples and a HEK293 cell disease model respectively

(**a**) RT-qPCR analysis shows that the levels of *ELL* and *ELL2* mRNA are increased significantly in frontal cortex of FTLD-TDP compared to normal frontal cortex controls. Bars represent mean (SD). (NS) not significant (both Two-tailed unpaired Student's *t* test and Two-tailed unpaired Mann-Whitney test for *ELL*; Two-tailed unpaired Student's *t* test for *ELL2*). Sample details are as indicated in Fig. 6b.
(**b**) Western blot analysis of ELL and ELL2 show that protein levels of ELL and ELL2 are not altered in HEK293 cells expressing GFP-TDP-43 compared to cells expressing GFP after 6d induction. *GAPDH* was used as internal control. *n*=4 biological replicates. Bars represent mean (SD). (NS) not significant (Two-tailed unpaired Student *t* test).

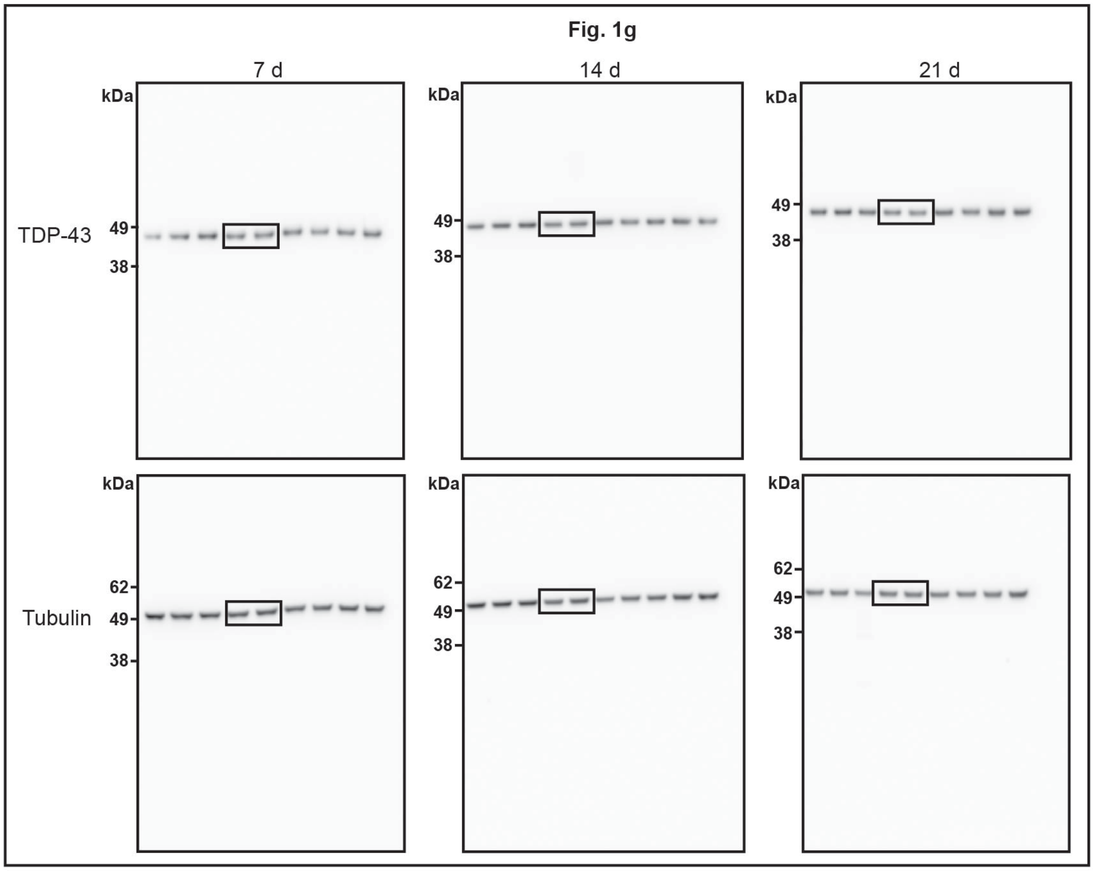

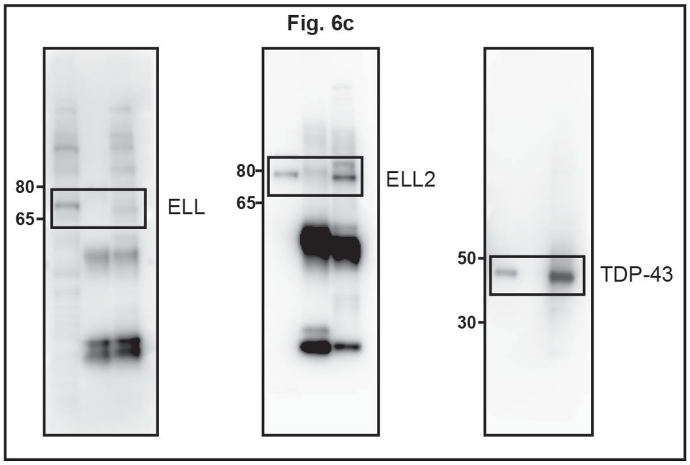

Supplementary Figure 7. The uncropped scans of western blots in Fig. 1g and Fig. 6c

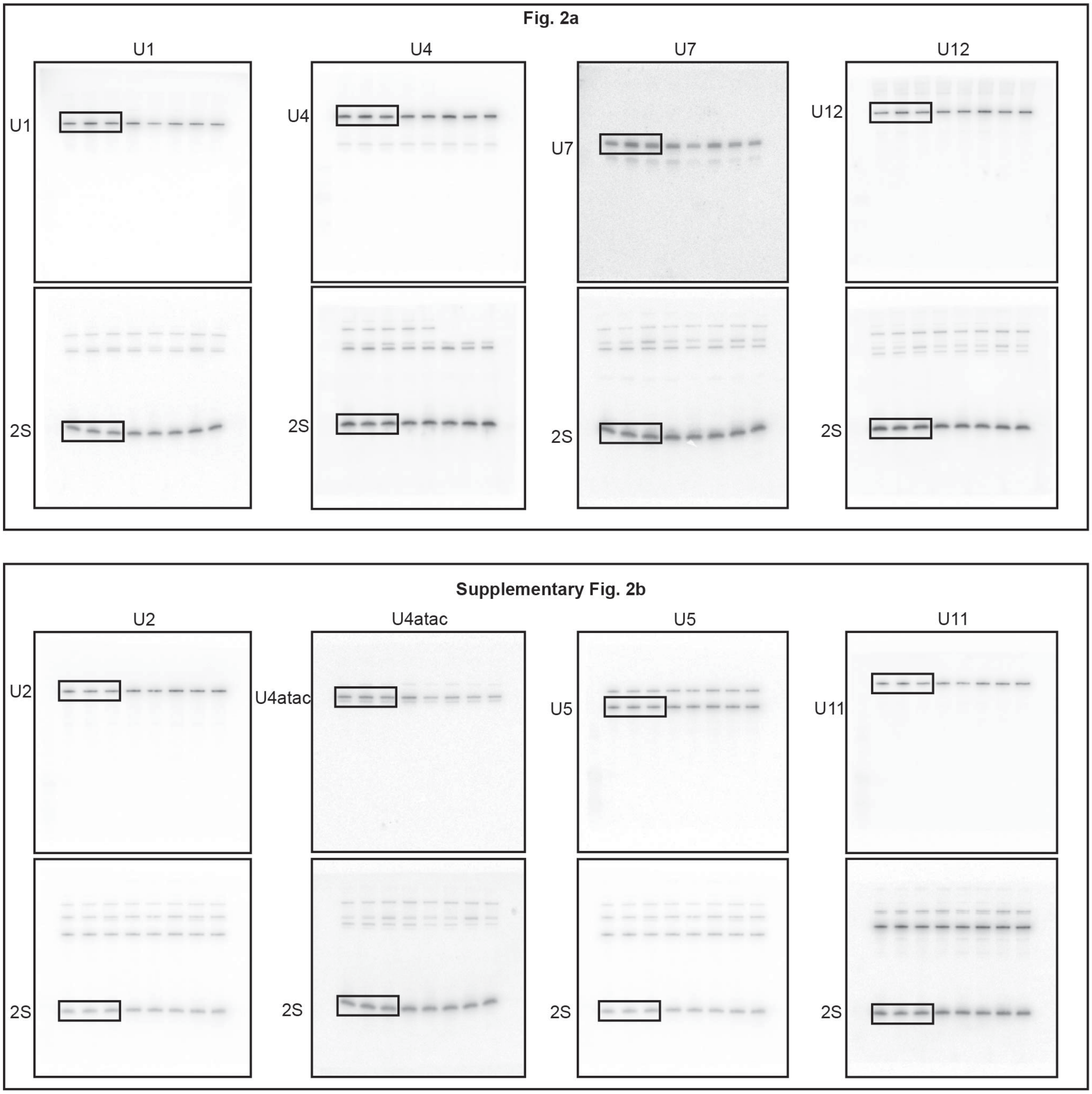

Supplementary Figure 8. The uncropped scans of small RNA Northern blots

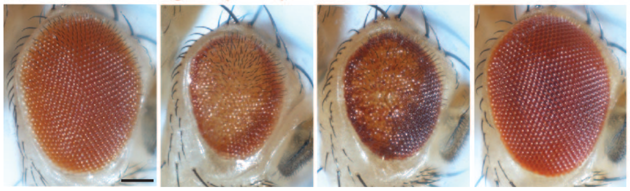

Supplementary Figure 9. Depletion of *Ell* suppresses the eye degeneration caused by G_4C_2 expansion

Expression of GGGGCC expansion causes eye degeneration, which is suppressed by *Ell* RNAi. Scale bars: 100 µm. Genotypes: Control is *gmr-GAL4(YH3)/+*. gmr>$(G_4C_2)_{48}$ is *UAS-*$(G_4C_2)_{48}$*/+; gmr-GAL4(YH3)/+*. gmr>$(G_4C_2)_{48}$+Control RNAi is *UAS-*$(G_4C_2)_{48}$*/+; gmr-GAL4(YH3)/UAS-Control.RNAi*^{JF01355}. gmr>$(G_4C_2)_{48}$+*Ell* RNAi is *UAS-*$(G_4C_2)_{48}$*/+; gmr-GAL4(YH3)/UAS-Ell.RNAi*^{HMS00277}.

Supplementary References

1. Smith ER*, et al.* The little elongation complex regulates small nuclear RNA transcription. *Mol Cell* **44**, 954-965 (2011).

2. Swain A*, et al.* Drosophila TDP-43 RNA-Binding Protein Facilitates Association of Sister Chromatid Cohesion Proteins with Genes, Enhancers and Polycomb Response Elements. *PLoS Genet* **12**, e1006331 (2016).

3. Elden AC*, et al.* Ataxin-2 intermediate-length polyglutamine expansions are associated with increased risk for ALS. *Nature* **466**, 1069-1075 (2010).

4. Tricoire H, Battisti V, Trannoy S, Lasbleiz C, Pret AM, Monnier V. The steroid hormone receptor EcR finely modulates Drosophila lifespan during adulthood in a sex-specific manner. *Mech Ageing Dev* **130**, 547-552 (2009).

5. Burguete AS, Almeida S, Gao FB, Kalb R, Akins MR, Bonini NM. GGGGCC microsatellite RNA is neuritically localized, induces branching defects, and perturbs transport granule function. *Elife* **4**, e08881 (2015).
