# Supplementary material for: Aberrant activation of non-coding RNA targets of transcriptional elongation complexes contributes to TDP-43 toxicity
Source: Nat Commun. 2018 Oct 23;9:4406. doi: 10.1038/s41467-018-06543-0 (PMC6199344; doi:10.1038/s41467-018-06543-0)
Supplement: Supplementary file 2 — Description of Additional Supplementary Files [file 41467_2018_6543_MOESM2_ESM.pdf]

Description of Additional Supplementary Files

File Name: Supplementary Data 1
Description: The lists of genes bound by TBPH, Lilli or both, derived from [1, 2]
